# Empagliflozin inhibits increased Na influx in atrial cardiomyocytes of patients with HFpEF

**DOI:** 10.1093/cvr/cvae095

**Published:** 2024-05-10

**Authors:** Maximilian Trum, Johannes Riechel, Elisa Schollmeier, Simon Lebek, Philipp Hegner, Kathrin Reuthner, Silvia Heers, Karoline Keller, Michael Wester, Susanne Klatt, Nazha Hamdani, Zdenek Provaznik, Christof Schmid, Lars Maier, Michael Arzt, Stefan Wagner

**Affiliations:** Department of Internal Medicine II, University Hospital Regensburg, Regensburg, Germany; Department of Internal Medicine II, University Hospital Regensburg, Regensburg, Germany; Department of Internal Medicine II, University Hospital Regensburg, Regensburg, Germany; Department of Internal Medicine II, University Hospital Regensburg, Regensburg, Germany; Department of Internal Medicine II, University Hospital Regensburg, Regensburg, Germany; Department of Internal Medicine II, University Hospital Regensburg, Regensburg, Germany; Department of Internal Medicine II, University Hospital Regensburg, Regensburg, Germany; Department of Internal Medicine II, University Hospital Regensburg, Regensburg, Germany; Department of Internal Medicine II, University Hospital Regensburg, Regensburg, Germany; Department of Internal Medicine II, University Hospital Regensburg, Regensburg, Germany; Department of Cellular and Translational Physiology, Ruhr-University Bochum, Bochum, Germany; Department of Cardiothoracic Surgery, University Hospital Regensburg, Regensburg, Germany; Department of Cardiothoracic Surgery, University Hospital Regensburg, Regensburg, Germany; Department of Internal Medicine II, University Hospital Regensburg, Regensburg, Germany; Department of Internal Medicine II, University Hospital Regensburg, Regensburg, Germany; Department of Internal Medicine II, University Hospital Regensburg, Regensburg, Germany

**Keywords:** Heart failure, HFpEF, Sodium, SGLT2i, Empagliflozin, Atrial remodelling

## Abstract

**Aims:**

Heart failure with preserved ejection fraction (HFpEF) causes substantial morbidity and mortality. Importantly, atrial remodelling and atrial fibrillation are frequently observed in HFpEF. Sodium–glucose cotransporter 2 inhibitors (SGLT2i) have recently been shown to improve clinical outcomes in HFpEF, and post-hoc analyses suggest atrial anti-arrhythmic effects. We tested if isolated human atrial cardiomyocytes from patients with HFpEF exhibit an increased Na influx, which is known to cause atrial arrhythmias, and if that is responsive to treatment with the SGTL2i empagliflozin.

**Methods and results:**

Cardiomyocytes were isolated from atrial biopsies of 124 patients (82 with HFpEF) undergoing elective cardiac surgery. Na influx was measured with the Na-dye Asante Natrium Green–2 AM (ANG-2). Compared to patients without heart failure (NF), Na influx was doubled in HFpEF patients (NF vs. HFpEF: 0.21 ± 0.02 vs. 0.38 ± 0.04 mmol/L/min (N = 7 vs. 18); *P* = 0.0078). Moreover, late I_Na_ (measured via whole-cell patch clamp) was significantly increased in HFpEF compared to NF. Western blot and HDAC4 pulldown assay indicated a significant increase in CaMKII expression, CaMKII autophosphorylation, CaMKII activity, and CaMKII-dependent Na_V_1.5 phosphorylation in HFpEF compared to NF, whereas Na_V_1.5 protein and mRNA abundance remained unchanged. Consistently, increased Na influx was significantly reduced by treatment not only with the CaMKII inhibitor autocamtide-2-related inhibitory peptide (AIP), late I_Na_ inhibitor tetrodotoxin (TTX) but also with sodium/hydrogen exchanger 1 (NHE1) inhibitor cariporide. Importantly, empagliflozin abolished both increased Na influx and late I_Na_ in HFpEF. Multivariate linear regression analysis, adjusting for important clinical confounders, revealed HFpEF to be an independent predictor for changes in Na handling in atrial cardiomyocytes.

**Conclusion:**

We show for the first time increased Na influx in human atrial cardiomyocytes from HFpEF patients, partly due to increased late I_Na_ and enhanced NHE1-mediated Na influx. Empagliflozin inhibits Na influx and late I_Na_, which could contribute to anti-arrhythmic effects in patients with HFpEF.

## Graphical Abstract

**Graphical abstract:**
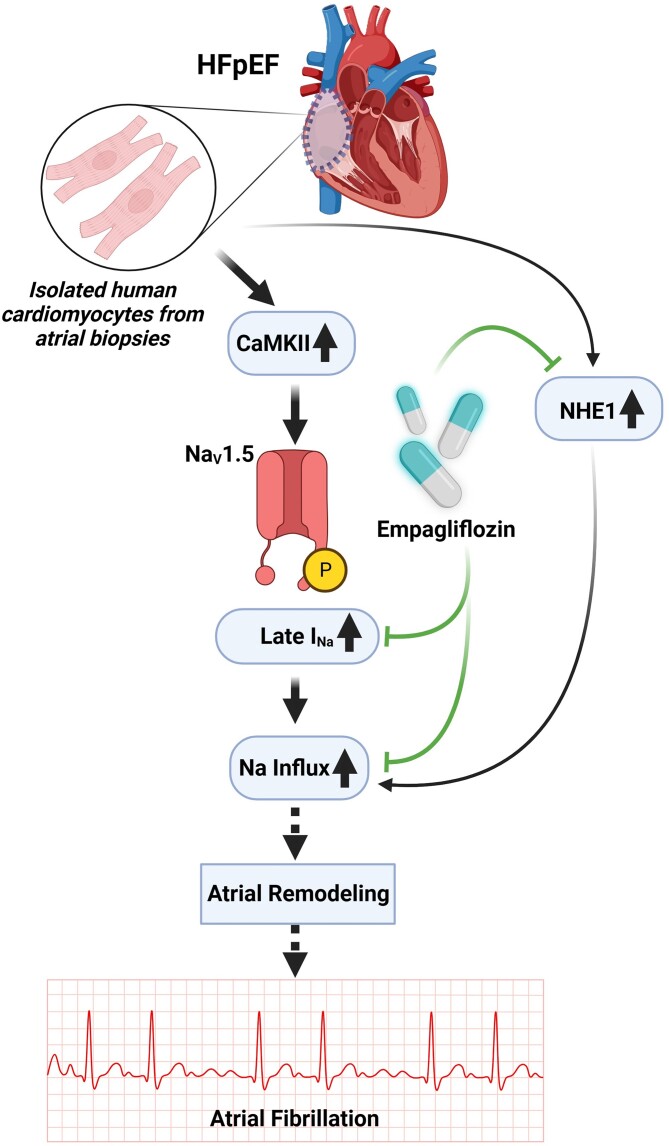

**Time of primary review: 20 days**

## 1. Introduction

As a result of landmark clinical trials, SGLT2 inhibitors (SGLT2i) have recently become a cornerstone in the treatment of patients with heart failure with reduced ejection fraction (HFrEF).^1,2^ Moreover, empagliflozin is the first drug to significantly reduce the combined risk of heart failure hospitalization (HHF) and cardiovascular death in patients with heart failure with preserved ejection fraction (HFpEF).^3^ Both forms of heart failure are associated with pronounced atrial remodelling and consequent increased risk of the development of atrial arrhythmias (e.g. atrial fibrillation, atrial flutter), which are frequent causes of heart failure hospitalizations.^4,5^ Na overload of cardiomyocytes is a hallmark of heart failure and contributes not only to systolic and diastolic dysfunction but also to ventricular and atrial arrhythmogenesis.^6–9^ Post-hoc analyses of clinical trials suggest a potential anti-arrhythmic effect of SGLT2i’s including a reduction of episodes of atrial arrhythmias including atrial fibrillation and atrial flutter.^10,11^ There are several lines of evidence for an effect of SGLT2i on Na handling in cardiomyocytes by inhibition of Ca/calmodulin-dependent protein kinase II (CaMKII),^12^ the late sodium current (late I_Na_)^13^ as well as the sodium/hydrogen exchanger 1 (NHE1),^14–17^ with the relevance of the latter one still being debated.^18,19^ However, in contrast to HFrEF, there are very few data on Na handling in human cardiomyocytes of patients with HFpEF, and the importance of the potential SGLT2i targets mentioned above for Na influx into cardiomyocytes is almost completely unknown in these patients. Intriguingly, an increase in CaMKII activity, which is associated with fundamental alterations in cardiomyocyte Na handling,^20^ was reported in a rat model of HFpEF,^21^ suggesting a potential role of this enzyme also in HFpEF patients.

With these considerations in mind, we here examined Na handling in isolated atrial cardiomyocytes from HFpEF patients compared with non-failing cardiomyocytes (NF) and evaluated the effect of the SGLT2i empagliflozin. Moreover, we differentiated the relative contribution of the above-mentioned Na entry pathways to Na influx in these cells.

## 2. Methods

### 2.1 Study design

This study is a cross-sectional, experimental study performed as part of the prospective observational study in cardiovascular patients undergoing cardiac surgery (NCT02877745). This study was performed in compliance with the Declaration of Helsinki (most recent revision in 2013) and approved by the local ethics committee (University of Regensburg, Bavaria, Germany; 15-238-101). Prior to inclusion in the study, each patient gave written informed consent. Patient data can be made available upon reasonable request only after informed consent has been given by each patient in the study.

Between May 2016 to July 2018 and February 2020 to October 2023, patients undergoing elective cardiac surgery for coronary and/or valvular heart disease at the University Hospital Regensburg were prospectively screened for eligibility. Inclusion criteria were elective coronary artery bypass graft (CABG) and/or valve surgery, age over 18 years, and written informed consent for study participation. Exclusion criteria were reduced ejection fraction and persistent/permanent atrial fibrillation (which might potentially confound the results due to prominent electrical and structural remodelling independent of heart failure). Prospectively, 200 patients were screened as eligible (see Supplementary material online, *Figure S1*). Of these patients, 16 were excluded because of permanent/persistent AF, 37 were excluded because of diagnosis of HFrEF/HFmrEF and 23 were excluded because of insufficient clinical data, resulting in 124 patients included in this study (see Supplementary material online, *Figure S1*). A diagnosis of HFpEF was made in 82 patients according to current guidelines, requiring a preserved left ventricular ejection fraction (LVEF ≥ 50%), signs and/or symptoms of heart failure, and objective evidence of cardiac abnormalities [elevated N-terminal pro-brain natriuretic peptide (NT-proBNP) levels (≥ 125 pg/nL) and/or structural abnormalities (left ventricular hypertrophy, left atrial enlargement, and/or diastolic dysfunction)]. Patients were defined as NF if they had a normal LVEF (≥ 50%) and did not fulfil the diagnostic criteria qualifying them for the diagnosis of HFpEF. After biopsies were obtained during surgery, they were transported in ice-cold Custodiol® solution [2 mmol/L butanedione monoxime (BDM)] for cardioplegia until analysis by blinded investigators in our laboratory.

### 2.2 Isolation of human atrial cardiomyocytes

Chunk isolation of human atrial cardiomyocytes from right atrial appendage biopsies was performed as previously described.^16,22^ Minced tissue was incubated at 37°C under continuous O_2_ bubbling in a spinner flask filled with Tyrode’s solution (in mmol/L: 100 NaCl, 10 KCl, 5 MgCl_2_, 0.02 CaCl_2_, 1.2 KH_2_PO_4_, 50 Taurine, 5 MOPS, 10 BDM, 20 glucose, pH 7.2) containing collagenase 1 and protease (type XXIV, 0.04%, Sigma). The solution was exchanged after 45 min for Tyrode’s solution containing collagenase 1 (protease omitted). This second digestion step was stopped by the addition of bovine calf serum (2%) and BDM (to 20 mmol/L) as soon as cardiomyocytes were visible in the solution. Next, cells were disaggregated by careful pipetting with a Pasteur pipette and subsequently centrifuged at 95 g for 10 min at room temperature. Finally, the cell pellet was resuspended in a storage medium (in mmol/L: 30 KCl, 10 KH_2_PO_4_, 1 MgCl_2_, 10 HEPES, 11 glucose, 20 taurine, 70 glutamic acid, 20 BDM, 2% BCS, and pH 7.4).^22,23^

### 2.3 Measurement of Na influx

The intracellular Na concentration was measured by loading cells with the Na-sensitive fluorescence indicator Asante Natrium Green–2 AM [ANG-2 (ION Biosciences LLC, San Marcos, USA)].^24,25^ Isolated atrial myocytes were immobilized on laminin-coated coverslips and incubated with 5 µM ANG-2 for 45 min at room temperature together with either empagliflozin (1 µM; Cayman Chemical, Ann Arbor, Michigan, USA), cariporide (10 µM; Sigma Aldrich, Hamburg, Germany), tetrodotoxin (TTX, 1 µM; Cayman Chemical, Ann Arbor, Michigan, USA), autocamtide-2-related inhibitory peptide (AIP, 1 µM, Sigma Aldrich, Hamburg, Germany) or DMSO (0,01%). Afterwards, coverslips were washed twice with Tyrode’s solution (in mmol/L: NaCl 140, KCl 4, MgCl_2_ 1, HEPES 5, glucose 10 and CaCl_2_ 1.8, pH 7.4 with NaOH) including the respective drug and given 10 min for dye de-esterification. Subsequently, the measurement chambers were mounted on a confocal microscope (Zeiss LSM 700; Jena, Germany). Atrial cardiomyocytes were field stimulated at 1 Hz and continuously superfused with Tyrode’s solution at 37°C. ANG-2 was excited at 488 nm, and emitted fluorescence F_545_ was acquired using a bandpass filter (536 nm, 40 nm bandwidth). Registered emission intensity was corrected by subtraction of background fluorescence.

F_545_ was calibrated using a sequence of calibration solutions with precisely set Na concentrations (in mmol/L: 0, 5, 10, and 20) generated by mixture of two solutions containing (in mmol/L) 140 NaCl, 10 HEPES, 1 EGTA, 5 strophantidin (specific Na/K-ATPase inhibitor), and 1 gramicidin D [ionophore (pH = 7.2 with TRIS: solution 1)] or 140 KCl instead of NaCl (solution 2), respectively (see Supplementary material online, *Figure S2*). Finally, the calibration curve was derived from a Boltzmann fit, and intracellular Na concentration ([Na]_i_) was calculated according to the following equation: $[Na{]}_{i}=\;\frac{F_{545\;}-\;F_{min}}{F_{max}-F_{545}}\times K_{D}$.

Na influx was measured according to a protocol modified by Despa *et al.*^26^ Cells were superfused with drug-containing Tyrode’s solution at 37°C until fluorescence reached a steady state (usually within 5–10 min). Afterwards, Na/K-ATPase was inhibited in myocytes by superfusion with potassium-free Tyrode’s solution (in mmol/L: NaCl 140, CsCl 4, MgCl_2_ 1, HEPES 5, glucose 10, and CaCl_2_ 1.8, pH 7.4 with NaOH) including the respective drug for 10 min. The resulting increase in intracellular Na can be used as a measure for transsarcolemmal Na influx. This Na influx was assessed as the mean slope of the increase in [Na]_i_ over time during Na/K-ATPase inhibition. To avoid bias, the measurement sequence of the different groups was randomly altered by the investigator, who was blinded to clinical data.

### 2.4 Late I_Na_ measurement

Ruptured whole-cell patch-clamp experiments in voltage-clamp mode were performed to assess late I_Na_ in isolated human atrial cardiomyocytes on laminin-coated recording chambers mounted on an inverted microscope (Zeiss Axio Observer).

Microelectrodes (3MΩ) were filled with (mmol/L) 95 CsCl, 40 Cs-glutamate, 10 NaCl, 0.92 MgCl2, 1 EGTA, 5 Mg-ATP, 0.3 Li-GTP, 0.36 CaCl2, 0.03 niflumic acid, 0.02 nifedipine, 0.004 strophanthidin, and 5 HEPES (pH 7.2, CsOH). The bath solution contained (mmol/L) 135 NaCl, 5 tetramethylammonium chloride, 4 CsCl, 2 MgCl2, 10 glucose, and 10 HEPES (pH 7.4, room temperature with CsOH). Cells were incubated with DMSO (= vehicle, 0.01%), empagliflozin (1 µM), or AIP (1 µM) for 30 min prior to patch rupture.

Resting membrane potential was held at −120 mV, and I_Na_ was elicited by depolarizing to −20 mV for 1000 ms. Late I_Na_ was quantified by integrating from 50 to 500 ms of the start of depolarizing and normalized to the membrane capacitance.

The experiments were performed at room temperature. Access resistance was usually <10 MΩ after patch rupture. Fast capacitance was compensated in a cell-attached configuration. Membrane capacitance and series resistance were compensated after patch rupture. Recordings began after 3 min of equilibration following patch rupture. Signals were filtered with 2.9 and 10 kHz Bessel filters and recorded with an EPC10 amplifier (HEKA Elektronik).

### 2.5 Western blot

Human right atrial appendages were homogenized in Tris buffer (in mmol/L: Tris–HCl 20, NaCl 200, NaF 20, Nonidet P-40 8.9 (Sigma Aldrich, Hamburg, Germany), phenylmethanesulfonylfluoride 18.3 (Sigma Aldrich, Hamburg, Germany), complete protease inhibitor cocktail (Roche diagnostics, Mannheim, Germany), and complete phosphatase inhibitor cocktail (Roche diagnostics, Mannheim, Germany). BCA assay (Pierce Biotechnology, Waltham, Massachusetts, USA) was used to determine protein concentration. After denaturation (30 min at 37°C for Na_V_1.5 or 5 min at 95°C for CaMKII and GAPDH at 500 rpm in 2% β-mercaptoethanol (Sigma Aldrich)), proteins were separated on 8% SDS-polyacrylamide gels and transferred to a nitrocellulose membrane (GE Healthcare). Membranes were incubated with primary antibodies (rabbit polyclonal anti-phospho-Na_V_1.5 (at serine 571, 1:1000, a kind gift from Prof. Dr. P. Mohler and Prof. Dr. T. Hund, The Ohio State University, Columbus, Ohio, USA), rabbit polyclonal anti-Na_V_1.5 (1:200, Alomone Labs, catalog number ASC-013), mouse monoclonal anti-CaMKII (1:1000, BD Biosciences, catalog number 611293), mouse monoclonal anti-phospho-CaMKII (1:1000, Affinity BioReagents, catalog number MA1-047), or mouse monoclonal anti-GAPDH (1:20000, Abcam, catalog number G8795)) overnight at 4°C. Secondary antibodies HRP-conjugated sheep anti-mouse IgG (1:3000 for anti-CaMKII, 1:5000 for anti-phospho-CaMKII, 1:30 000 for anti-GAPDH, GE Healthcare, catalog number NA931VS) and donkey anti-rabbit IgG (1:5000 for anti-phospho-Na_V_1.5 and for anti-Na_V_1.5, GE Healthcare, catalog number NV934 V) were incubated for 1 h at room temperature.

### 2.6 CaMKII activity assay

For the determination of CaMKII activity, a highly specific CaMKII activity assay was applied, as previously described.^27,28^ In brief, GST-HDAC4 419-670 fusion protein containing a CaMKII activity-dependent binding domain was used for CaMKII pulldown from homogenized samples. By washing with sodium phosphate-buffered solution (PBS), unbound (and thus inactive) CaMKII was removed. Finally, bound (activated) CaMKII was assessed for each pulldown experiment and normalized to the input bait GST-HDAC4 in the sample.

Detection of the protein bands was enabled by incubation with Immobilon™ Western Chemiluminescent HRP Substrate (Millipore, Darmstadt, Germany) for 5 min at room temperature. Afterwards, the bands were developed onto Super XR-N X-ray films (Fujifilm, Düsseldorf, Germany) and scanned using ChemiDoc™ MP Imaging System (Bio-Rad, Feldkirchen, Germany). ImageJ was used to analyse mean densitometric values.

### 2.7 Quantification of *SCN5A* mRNA expression

mRNA was extracted from right atrial biopsies using the RNeasy Mini Kit (Qiagen, Hilden, Germany) according to the manufacturer’s recommendation. cDNA was transcribed from 1 µg RNA using random primers, PCR nucleotide mix, RNasin ribonuclease inhibitor, reverse transcriptase, and reverse transcriptase 5 × reaction buffer (Promega GmbH, Walldorf, Germany) for 1 h at 37°C. mRNA Abundance of Na_V_1.5 (*SCN5A*; Assay ID: Hs00165693_m1*)* and β-actin (*ACTB*; Assay ID: Hs99999903_m1) was measured using the TaqMan Gene Expression (Thermo Fisher Scientific GmbH, Dreieich, Germany) detection method. For relative mRNA expression analysis according to the comparative threshold cycle (Ct) relative quantification analysis method,^29^ β-actin was used as the housekeeper gene.

### 2.8 Statistics

All experiments were performed and analysed blinded to the clinical data. Experimental data are presented as mean values per patient, unless indicated otherwise, ± standard error of the mean (SEM). Clinical data are presented as total number of patients (with relative proportion), mean ± standard deviation (SD, if normally distributed), or mean ± interquartile range (IQR, if not normally distributed), as appropriate. If more than one observation was acquired in a single patient, the mean of these observations was calculated first to obtain a single value for each patient. Statistics were always based on the number of patients. For comparison of normally distributed data between two groups, Student’s *t*-test was used. For comparison of paired data with more than two groups that were normally distributed, mixed-effects analysis with Holm-Sidak’s post-hoc correction or one-way repeated measures ANOVA with Holm-Sidak’s post-hoc correction was applied. For some experiments, univariate or multivariate linear regression was conducted. Two models were applied for multivariate linear regression analysis. They included existing HFpEF, age, sex, and BMI (Model I) or existing HFpEF, age, sex, BMI, AF, diabetes, arterial hypertension, NT-proBNP, and eGFR (Model II) as potential confounding clinical parameters.

Differences in patients’ clinical baseline characteristics were analysed using Pearson’s χ^2^ test for categorical data and Student’s *t*-test or Wilcoxon rank-sum test for continuous data, respectively. All these tests were performed using GraphPad Prism 10 or Stata/MP 13.0. Two-sided *P*-values below 0.05 were considered statistically significant.

## 3. Results

### 3.1 Patient characteristics

Right atrial tissue from 124 patients undergoing elective cardiac surgery for coronary and/or valvular heart disease was analysed in this study (*Table 1*). Overall, the patients represent a typical cohort of patients with heart disease. They were predominantly male patients (83% vs. 80%; NF vs. HFpEF, *P* = 0.70, *Table 1*) at an average age of 63.4 ± 8.7 and 68.6 ± 8.1 years, respectively (NF vs. HFpEF, *P* = 0.001, *Table 1*). Nineteen to 27% of the patients suffered from diabetes mellitus (NF vs. HFpEF, *P* = 0.34, *Table 1*), which was for the most part adequately controlled (HbA1c 5.95 ± 0.77% vs. 5.87 ± 0.98%, NF vs. HFpEF, *P* = 0.57, *Table 1*). Arterial hypertension was diagnosed in 80–86% of patients (NF vs. HFpEF; *P* = 0.68, *Table 1*). The body mass index (BMI) was similar in both groups (29.0 ± 4.4 kg/m^2^ vs. 27.91 ± 4.6 kg/m^2^, *P* = 0.18, *Table 1*). While the proportion of patients suffering from severe aortic valve stenosis was higher in the HFpEF group (0% vs. 19%, NF vs. HFpEF, *P* = 0.006, *Table 1*), almost all NF patients were diagnosed with coronary artery disease (95% vs. 78%, *P* = 0.014, *Table 1*). In conformity with the diagnostic criteria, HFpEF patients more frequently demonstrated enlarged left atria (29% vs. 55%, NF vs. HFpEF, *P* = 0.010, *Table 1*) as well as diastolic dysfunction (22% vs. 92%, NF vs. HFpEF, *P* < 0.001, *Table 1*). Median NT-proBNP levels were significantly increased in HFpEF patients (80.9 [95% CI: 50; 119] pg/mL vs. 437.0 [95% CI: 240; 811] pg/mL, *P* < 0.001, *Table 1*) and 15% of them suffered from paroxysmal atrial fibrillation (vs. 0% in NF, *P* = 0.009, *Table 1*). Kidney function appeared worse in HFpEF patients compared to NF patients (eGFR 82.0 ± 16.8 vs. 71.1 ± 20.1 mL/min/1.73 m^2^, *P* = 0.003, *Table 1*). There was no difference regarding treatment with ACEi, AT1-receptor antagonists, β-blockers, calcium channel blockers, mineralocorticoid receptor antagonists, ranolazine, or diuretics between groups. None of the patients were treated with an angiotensin receptor neprilysin inhibitor (ARNI). One patient in the NF group and only two patients in the HFpEF group were pre-treated with an SGLT2i (2% vs. 2%, *P* = 0.98), which was periprocedurally paused.

**Table 1:** Baseline characteristics

	NF (*N* = 42)	HFpEF (*N* = 82)	*P* value
Age (years), mean ± SD	63.4 ± 8.7 (*n* = 42)	68.6 ± 8.1 (*n* = 82)	**0.001** ^t^
Male gender, *n* (%)	35 (83)	66 (80)	0.70^chi^
Body mass index (kg/m^2^), mean ± SD	29.0 ± 4.4 (*n* = 42)	27.9 ± 4.6 (*n* = 82)	0.18^t^
Coronary artery disease, *n* (%)	40 (95)	64 (78)	**0.01** ^chi^
Severe aortic valve stenosis, *n* (%)*	0 (0)	15 (19)	**0.006** ^chi^
Hypertension, *n* (%)	34 (81)	68 (83)	0.79^chi^
Diabetes mellitus, *n* (%)	8 (19)	22 (27)	0.34^chi^
HbA1c (%), mean ± SD	5.95 ± 0.77 (*n* = 39)	5.87 ± 0.98 (*n* = 79)	0.68^t^
Hyperlipidemia, *n* (%)	27 (64)	52 (63)	0.92^chi^
eGFR (mL/min/1.73 m^2^), mean ± SD	82.0 ± 16.8 (*n* = 42)	71.1 ± 20.1 (*n* = 82)	**0.003** ^t^
LVEF (%), mean ± SD	60.0 ± 5.0 (*n* = 42)	59.6 ± 6.1 (*n* = 82)	0.75^t^
Dilated left atrium, *n* (%)**	10 (29)	39 (55)	**0.01** ^chi^
Diastolic dysfunction, *n* (%)***	8 (22)	55 (92)	**<0.001** ^chi^
Paroxysmal atrial fibrillation, *n* (%)	0 (0)	12 (15)	**0.009** ^chi^
NT-pro BNP (pg/mL), median (IQR)	80.9 (50, 119) (*n* = 40)	437 (240, 811) (*n* = 78)	**<0.001** ^w^
NYHA functional status, *n* (%)			**<0.001** ^chi^
I	22 (71)	3 (4)	
II	9 (29)	60 (74)	
III	0 (0)	17 (21)	
IV	0 (0)	1 (1)	
ACE-inhibitors, *n* (%)	24 (57)	42 (51)	0.53^chi^
AT1-receptor antagonists, *n* (%)	7 (17)	19 (23)	0.40^chi^
ARNI, *n* (%)	0 (0)	0 (0)	
Beta blockers, *n* (%)	30 (71)	50 (61)	0.25^chi^
Spironolactone, *n* (%)	0 (0)	3 (4)	0.21^chi^
Calcium channel blocking agents, *n* (%)	9 (21)	24 (29)	0.35^chi^
Ranolazine, *n* (%)****	1 (3)	0 (0)	0.15^chi^
Loop diuretics, *n* (%)	6 (14)	21 (26)	0.15^chi^
Thiazide diuretics, *n* (%)	11 (27)	23 (28)	0.89^chi^
SGLT2 inhibitor, *n* (%)	1 (2)	2 (2)	0.98^chi^

IQR indicates interquartile range, which lies between the 25th and 75th percentiles. The bold values are the parameters that show a statistically significant correlation. Abbreviations: NF, non-failing; HFpEF, heart failure with preserved ejection fraction; eGFR, estimated glomerular filtration rate; LVEF, left ventricular ejection fraction; NT-proBNP, N-terminal pro-brain natriuretic peptide; NYHA, New York Hear Association; ACE, angiotensin-converting enzyme; AT1, angiotensin receptor I; ARNI, angiotensin receptor neprilysin inhibitor; SGLT2, sodium–glucose contransporter-2; ^t^ = Student’s *t*-test; ^chi^ = Pearson’s χ^2^ test; ^w^ = Wilcoxon rank-sum test. Divergent patient number from total: *Severe aortic valve stenosis (NF: *n* = 24/30); ** Dilated left atrium (NF: *n* = 35/42; HFpEF: 71/82); *** Diastolic dysfunction (NF: *n* = 37/42; HFpEF: *n* = 60/82); **** Ranolazine (NF: *n* = 35/42; HFpEF: *n* = 73/82).

### 3.2 Atrial cardiomyocyte Na influx is increased in HFpEF

**Figure 1:**
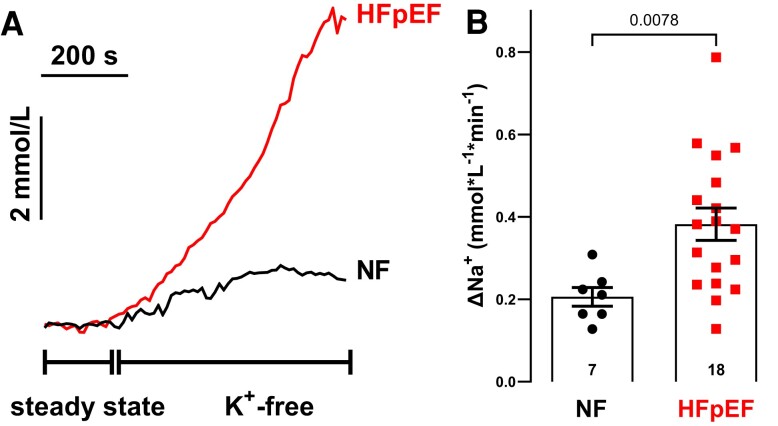
Increased Na influx into isolated atrial cardiomyocytes of HFpEF patients. Original registrations of Na influx in isolated human atrial cardiomyocytes following inhibition of Na/K-ATPase by superfusion with K-free solution (*A*). Na influx was calculated as mean slope of the increase in [Na]_i_ over time and derived values are illustrated as scatter plot (*B*). Na influx is almost doubled in isolated cardiomyocytes of HFpEF patients compared to NF patients (*P* = 0.0078, mixed-effects analysis with Holm-Sidak’s post-hoc correction).

In a first step, we investigated, if transsarcolemmal Na influx into cardiomyocytes is increased in HFpEF patients. Therefore, the intracellular Na concentration was measured in isolated atrial cardiomyocytes from NF and HFpEF patients by ANG-2 fluorescence. The increase in [Na]_i_ after inhibition of Na/K-ATPase (by superfusion with K-free solution) can be used as a measure of transsarcolemmal Na influx. Intriguingly, during steady-state electrical stimulation at 1 Hz, Na influx was almost doubled in HFpEF compared to NF cardiomyocytes (NF vs. HFpEF: 0.21 ± 0.02 vs. 0.38 ± 0.04 mmol/L/min (N = 7 vs. 18); *P* = 0.0078; *Figure 1A* and *B*), and remained significantly increased after correction for clinical confounders (multivariate linear regression: model I: B [95% CI]: 0.003 [0.0003; 0.006], *R*^2^ = 0.257, *P* = 0.033; model II: B [95% CI]: 0.003 [0.0001; 0.007], *R*^2^ = 0.463, *P* = 0.047, *Table 2*).

**Table 2:** Linear regression analysis for Na influx

	Simple Linear Regression Analysis		Multiple Linear Regression Analysis			
			Model I	*R* ^2^ 0.257	Model II	*R* ^2^ 0.463
Variable	B (95% CI)	*P* value	B (95% CI)	*P* value	B (95% CI)	*P* value
HFpEF	0.0029 (0.0007; 0.0052)	**0**.**013**	0.0029 (0.0003; 0.0055)	**0**.**033**	0.0033 (0.00006; 0.0066)	**0**.**047**
Age	0.00003 (−0.0001; 0.0002)	0.719	0.00004 (−0.0001; 0.0002)	0.605	0.00004 (−0.0001; 0.0002)	0.550
Male gender	−0.0014 (−0.0039; 0.0011)	0.245	−0.0002 (−0.0029; 0.0025)	0.867	−0.0006 (−0.0037; 0.0024)	0.671
BMI	6.58 × 10^−6^ (−0.0002; 0.0003)	0.957	0.00004 (−0.0002; 0.0003)	0.738	−0.00002 (−0.0003; 0.0003)	0.872
AF	0.0021 (−0.0004; 0.0047)	0.095			0.0011 (−0.0030; 0.0052)	0.573
Diabetes	0.0019 (−0.0009; 0.0046)	0.181			0.0030 (−0.0002; 0.0061)	0.061
Art. Hypertension	0.0002 (−0.0022; 0.0027)	0.841			−0.0015 (−0.0043; 0.0013)	0.269
NT-proBNP	3.44 × 10^−7^ (−2.08 × 10^−6^; 2.76 × 10^−6^)	0.772			−2.0 × 10^−6^ (−5.6 × 10^−6^; 1.6 × 10^−6^)	0.245
eGFR	−0.00001 (−0.00007; 0.00004)	0.645			−6.9 × 10-6 (−0.00008; 0.00007)	0.848

Model I accounts for age, gender, and BMI. Model II accounts for age, male gender, BMI, AF, diabetes, arterial hypertension, NT-proBNP, and eGFR. The bold values are the parameters that show a statistically significant correlation. Abbreviations: HFpEF, heart failure with preserved ejection fraction; BMI, body mass index (in kg/m^2^); AF, atrial fibrillation; NT-proBNP, N-terminal pro-brain natriuretic peptide (in pg/mL); eGFR, estimated glomerular filtration rate (in mL/min/1.73 m^2^).

### 3.3 Na_V_1.5 phosphorylation and late I_Na_ are increased in atrial cardiomyocytes of HFpEF patients

**Figure 2:**
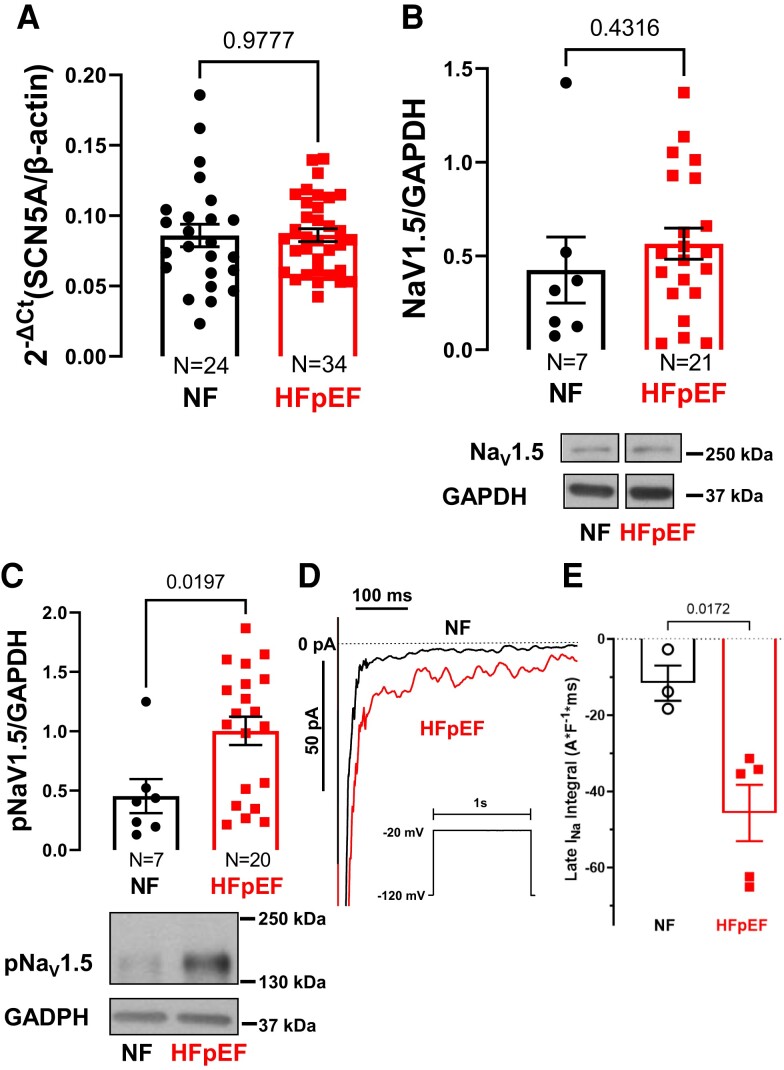
Increased Na_V_1.5 phosphorylation in atrial cardiomyocytes of HFpEF patients is associated with increased late I_Na_. Na_V_1.5 mRNA expression (gene: *SCN5A* [relative to β-actin as housekeeper]) was similar in NF and HFpEF cardiomyocytes (*P* = 0.9777, *t*-test) (*A*). Mean densitometric data (top) and representative original Western blot (bottom) of Na_V_1.5 (*B*) and pNa_V_1.5 (*C*). Compared to control patients (NF), there was a significant increase in the phosphorylation of Na_V_1.5 at S571 in atrial cardiomyocytes from patients with HFpEF (*P* = 0.0197, *t*-test), while Na_V_1.5 protein levels were unaltered (*P* = 0.4316, *t*-test). Original registrations of late I_Na_ (*D*) and mean values of late I_Na_ integrals (*E*) in atrial cardiomyocytes of HFpEF and NF patients measured by whole-cell patch clamp (voltage protocol is shown in the inset). In line with increased NaV1.5 phosphorylation late I_Na_ was significantly increased in atrial cardiomyocytes of HFpEF patients compared to NF patients (N = 3 vs. 5, *P* = 0.0172, *t*-test). *N*, number of patients.

Since cardiac Na_V_1.5 is a major contributor to transsarcolemmal Na influx, we performed Western blot and PCR to analyse the expression/mRNA levels of the cardiac sodium channel Na_V_1.5 in atrial tissue of patients without heart failure (NF) and patients with HFpEF. Both *SCN5A* mRNA levels (NF vs. HFpEF: 0.09 ± 0.007 vs. 0.09 ± 0.0051 (N = 24 vs. 34); *P* = 0.98, *Figure 2A*) and Na_V_1.5 protein expression (NF vs. HFpEF: 0.43 ± 0.18 A.U. vs. 0.57 ± 0.08 A.U. (N = 7 vs. 21), *P* = 0.43, *Figure 2B*) were similar in both groups. However, there was a significant increase in CaMKII-dependent Na_V_1.5 channel phosphorylation at serine 571 in HFpEF compared to NF (NF vs. HFpEF: 0.45 ± 0.14 vs. 1.00 ± 0.12 (N = 7 vs. 20); *P* = 0.02, *Figure 2C*). Importantly, in a multivariate linear regression analysis accounting for the demographic parameters age and BMI (gender was omitted from the analysis since coincidentally all analysed patients were male), HFpEF remained the only significant and independent predictor for increased Na_V_1.5 phosphorylation (B [95% CI]: 0.495 [0.005; 0.985], *R*^2^ = 0.242, *P* = 0.048, Model I, Supplementary material online, *Table S1*). When the model was further extended by the potential confounders AF, diabetes, arterial hypertension, NT-proBNP level, and eGFR, a trended positive correlation between HFpEF and Na_V_1.5 phosphorylation persisted without reaching statistical significance (B [95% CI]: 0.559 [−0.137; 1.254], *R*^2^ = 0.368, *P* = 0.107, Model II, Supplementary material online, *Table S1*). Na_V_1.5 phosphorylation is known to result in an increase in late I_Na_.^20^ Consistently, late I_Na_ is significantly increased in atrial cardiomyocytes of HFpEF patients compared to NF patients (NF vs. HFpEF; −11.60 ± 4.63 A*ms*F^−1^ vs. −45.69 ± 7.41 A*ms*F^−1^, N = 3 vs. N = 5, *P* = 0.017, *Figure 2D* and *E*).

### 3.4 CaMKII expression, phosphorylation, and activity are increased in atrial cardiomyocytes of HFpEF patients

**Figure 3:**
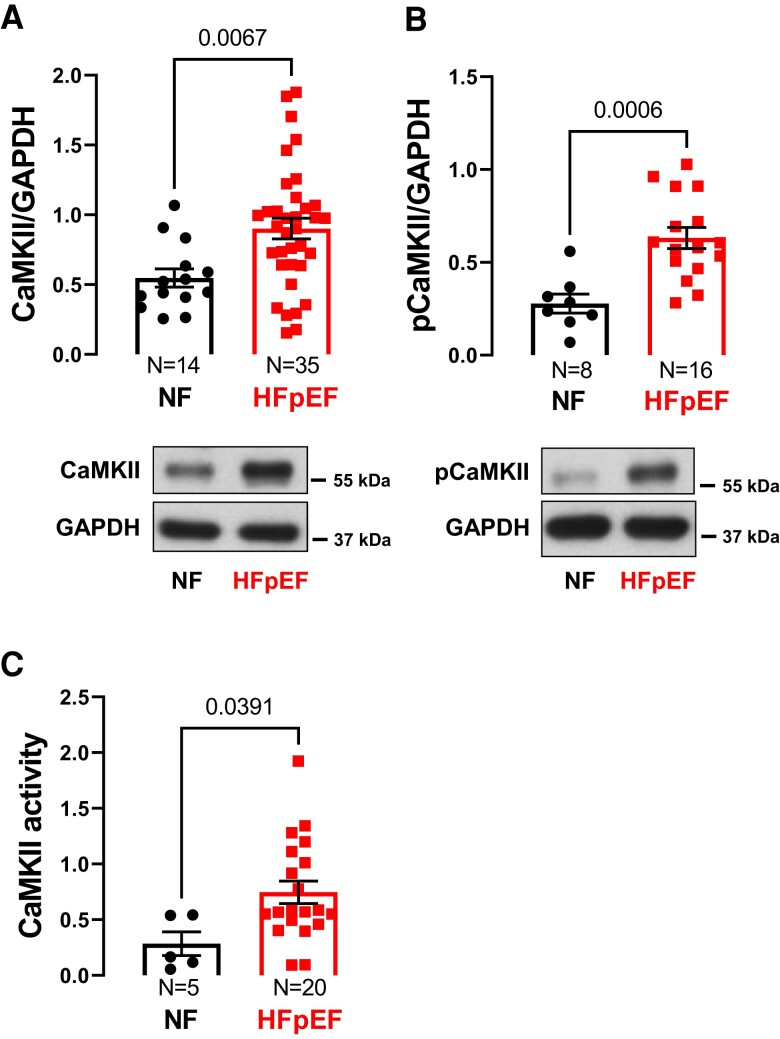
Increased CaMKII expression, autophosphorylation, and activity in atrial cardiomyocytes of HFpEF patients. Mean densitometric data (top) and representative original Western blot (bottom) of CaMKII expression (*A*) and phosphorylation (*B*) showing a significant increase in CaMKII expression (*P* = 0.0067, *t*-test) as well as CaMKII autophosphorylation at T287 (*P* = 0.0006, *t*-test) in atrial cardiomyocytes of HFpEF patients compared to NF patients. Accordingly, CaMKII activity assessed by HDAC4 pulldown assay was also enhanced in HFpEF patients (*P* = 0.0391, *t*-test) (*C*). N, number of patients.

As hyperphosphorylation of Ser571 at Na_V_1.5 is known to be CaMKII-dependent, we next investigated CaMKII expression and activity in the atrial tissue of NF and HFpEF patients. Indeed, CaMKII expression was significantly increased in the atrial myocardium of patients with HFpEF (NF vs. HFpEF: 0.55 ± 0.06 A.U. vs. 0.90 ± 0.07 A.U. (N = 14 vs. 35); *P* = 0.007, *Figure 3A*). This significant positive correlation between HFpEF and increased CaMKII expression remained significant even after accounting for many clinical covariates (multivariate linear regression: model I: B [95% CI]: 0.382 [0.079; 0.685], *R*^2^ = 0.156, *P* = 0.015; model II: B [95% CI]: 0.499 [0.150; 0.849], *R*^2^ = 0.255, *P* = 0.006, Supplementary material online, *Table S2*).

Besides CaMKII expression, CaMKII autophosphorylation at Thr287, a measure of CaMKII activity, was also markedly increased in atrial myocytes of HFpEF patients (NF vs. HFpEF: 0.28 ± 0.05 A.U. vs. 0.63 ± 0.06 A.U. (N = 8 vs. 16); *P* < 0.001; *Figure 3B*), and this correlation also remained significant after accounting for the above-mentioned clinical covariates (multivariate linear regression: model I: B [95% CI]: 0.295 [0.074; 0.515], *R*^2^ = 0.462, *P* = 0.011; Model II: B [95% CI]: 0.339 [0.122; 0.556], *R*^2^ = 0.702, *P* = 0.005, Supplementary material online, *Table S3*). In line with increased CaMKII expression and autophosphorylation, CaMKII activity (assessed by HDAC4 pull down) was also significantly increased in the atria of HFpEF patients (NF vs. HFpEF: 0.28 ± 0.11 A.U. vs. 0.75 ± 0.10 A.U. (N = 5 vs. 20); *P* = 0.039; *Figure 3C*).

### 3.5 Inhibition of CaMKII, late I_Na_, NHE1 as well as treatment with empagliflozin reduces Na influx in HFpEF

**Figure 4:**
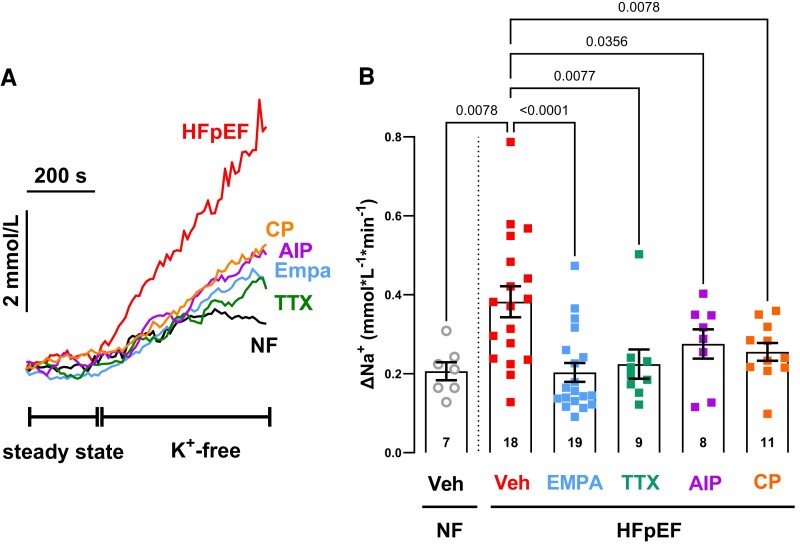
Increased Na influx in HFpEF patients is reduced by treatment with empagliflozin, TTX, AIP, and CP. Original registrations (*A*) and mean values per patient (*B*) of Na influx in isolated human atrial cardiomyocytes following inhibition of Na/K-ATPase by superfusion with K-free solution. Treatment of cardiomyocytes with empagliflozin resulted in a significant reduction of Na influx into HFpEF cardiomyocytes (*P* < 0.0001, mixed-effects analysis with Holm-Sidak’s post-hoc correction), similar to the treatment with the late I_Na_ inhibitor TTX (*P* = 0.0077, mixed-effects analysis with Holm-Sidak’s post-hoc correction), the CaMKII inhibitor AIP (*P* = 0.0356, mixed-effects analysis with Holm-Sidak’s post-hoc correction) and the NHE1 inhibitor cariporide (CP [*P* = 0.0078, mixed-effects analysis with Holm-Sidak’s post-hoc correction]). Numbers at the bottom of the bars represent the respective number of patients analysed.

Consistent with our protein data, treatment of atrial cardiomyocytes of HFpEF patients with the specific late I_Na_ inhibitor TTX (1 µM) significantly reduced Na influx into these cells (HFpEF + vehicle vs. HFpEF + TTX, 0.38 ± 0.04 mmol/L/min (N = 18) vs. 0.22 ± 0.04 mmol/L/min (*N* = 9); *P* = 0.0077, *Figure 4A* and *B*). Similar results could be achieved by treatment with the specific CaMKII inhibitor AIP (1 µM) (0.28 ± 0.04 mmol/L/min (N = 8); *P* = 0.0356 vs. HFpEF + vehicle, *Figure 4A and B*). We and others previously reported that SGLT2i inhibit CaMKII, late I_Na_, and NHE1 all of which are potentially centrally involved in cardiomyocyte Na handling.^12–17,30^ We, therefore, exposed ANG-2-loaded atrial myocytes to empagliflozin (1 µM, 60 min pre-incubation). Empagliflozin, similarly to AIP and TTX, completely abolished the increase in Na influx into cardiomyocytes from HFpEF patients (HFpEF + vehicle vs. HFpEF + empagliflozin: 0.38 ± 0.04 vs. 0.20 ± 0.02 mmol/L/min [*N* = 18 vs. 19]; *P* < 0.0001, *Figure 4A* and *B*). Of note, the NHE1 inhibitor cariporide (10 µM) also caused a significant decrease in Na influx (0.26 ± 0.02 mmol/L/min (N = 11); *P* = 0.0078 vs. HFpEF + vehicle, *Figure 4A* and *B*). Furthermore, combination of TTX and empagliflozin resulted in a more pronounced inhibition of Na influx than treatment with TTX alone (HFpEF + TTX vs. HFpEF + TTX + empagliflozin, 0.22 ± 0.04 mmol/L/min (N = 9) vs. 0.11 ± 0.01 mmol/L/min (N = 6); *P* = 0.031, Supplementary material online, *Figure S3A*). Neither empagliflozin (NF + vehicle vs. NF + empagliflozin, 0.21 ± 0.02 mmol/L/min (N = 7) vs. 0.26 ± 0.04 mmol/L/min (N = 10), *P* = 0.474) nor TTX (0.21 ± 0.04 mmol/L/min (N = 7), *P* = 0.969 vs. NF + vehicle) reduced Na influx in atrial cardiomyocytes of NF patients (see Supplementary material online, *Figure S3B*).

### 3.6 Increased late I_Na_ in atrial cardiomyocytes of HFpEF patients is normalized by empagliflozin

As the NHE inhibitor cariporide was also able to significantly reduce Na influx (see *Figure 4*) and addition of empagliflozin on top of TTX mediated a further decrease in Na influx (see Supplementary material online, *Figure S3A*), we set out to directly measure late I_Na_ to further discern the potential effect of empagliflozin treatment on this current in atrial cardiomyocytes.

**Figure 5:**
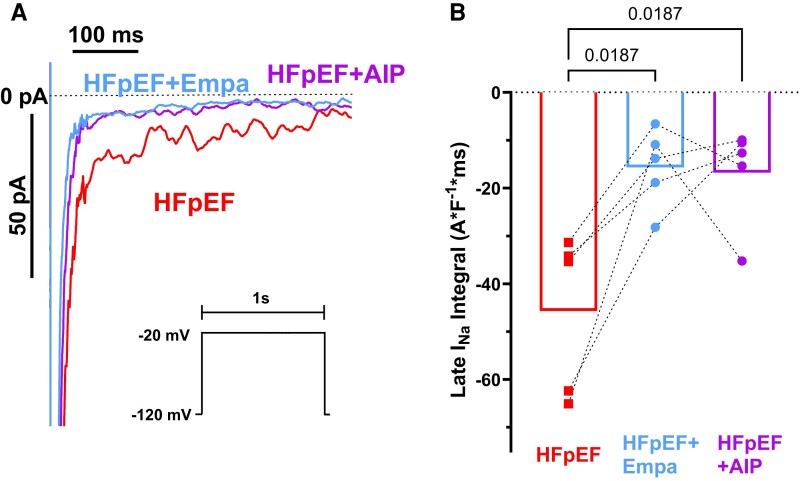
Increased late I_Na_ in atrial cardiomyocytes of HFpEF patients is normalized by treatment with empagliflozin as well as AIP. (*A*) Original registrations of late I_Na_ and (*B*) mean values of late I_Na_ integrals in atrial cardiomyocytes of HFpEF patients treated with vehicle solution, empagliflozin, or the CaMKII inhibitor AIP measured by whole-cell patch clamp (voltage protocol is shown in the inset). Empagliflozin as well as the CaMKII inhibitor AIP significantly reduced late I_Na_ (*P* = 0.0187 vs. HFpEF + vehicle, repeated measures one-way ANOVA with Holm-Sidak’s post-hoc correction).

Treatment of atrial cardiomyocytes from HFpEF patients with empagliflozin resulted in a reduction of late I_Na_ to the level of NF patients (HFpEF + vehicle vs. HFpEF + empagliflozin; −45.69 ± 7.41 A*ms*F^−1^ vs. −15.67 ± 3.7 A*ms*F^−1^, N = 5 vs. N = 5, *P* = 0.0187, *Figure 5A* and *B*). This effect was comparable to treatment with the specific CaMKII inhibitor AIP (HFpEF + vehicle vs. HFpEF + AIP; −45.69 ± 7.41 A*ms*F^−1^ vs. −16.75 ± 4.7 A*ms*F^−1^, N = 5 vs. N = 5, *P* = 0.0187, *Figure 5A* and *B*), suggesting a role of CaMKII for increased late I_Na_ in atrial cardiomyocytes of HFpEF patients.

## 4. Discussion

This is the first study to demonstrate increased Na influx in human atrial cardiomyocytes from HFpEF patients. Furthermore, we propose a mechanistic link between increased Na influx and increased CaMKII activity with enhanced CaMKII-dependent Na_V_1.5 phosphorylation leading to stimulation of late I_Na_. Both selective Na channel blockade with TTX and CaMKII inhibition with AIP but also NHE1 inhibition with cariporide abolished increased Na influx. Importantly, treatment with empagliflozin normalized increased late I_Na_ in atrial cardiomyocytes of HFpEF patients and normalized overall Na influx, which could contribute to the cardioprotective effects of this drug in these patients.

### 4.1 Late I_Na_, NHE1, and CaMKII contribute to increased Na influx in atrial cardiomyocytes of HFpEF patients

It is widely accepted that cardiomyocytes from HFrEF patients are characterized by cytosolic Na ([Na]_i_) and Ca ([Ca]_i_) overload, due in large part to deleterious CaMKII-dependent changes in excitation-contraction coupling as well as excitation-transcription coupling.^20,28,31–34^ For instance, CaMKII-dependent phosphorylation of Na_V_1.5 results in enhanced late I_Na_,^20,35^ an important pathway of Na entry into diseased cardiomyocytes, as seen in patients with HFrEF and persistent/permanent atrial fibrillation.^6,23^ Noteworthy, in animal models (Dahl salt-sensitive rats, rats with cardiorenal-induced HFpEF after subtotal nephrectomy), long-term treatment with the late I_Na_ inhibitor ranolazine and the NCX inhibitors ORM-11035 and SEA0400, respectively, was able to improve diastolic function and to attenuate cardiac remodelling,^7,36,37^ suggesting a possible dysregulation of Na and Ca handling also in HFpEF. Noteworthy, it was previously shown in a mouse model expressing a human Na_V_1.5 variant harbouring a mutation in the anaesthetic-binding site, resulting in incomplete Na channel inactivation, that increased Na influx via late I_Na_ alone was sufficient to drive structural (including atrial dilatation, and fibrosis) and electrophysiological alterations leading to spontaneous episodes of AF.^38^ Whether such alterations of Na handling also play a role in atria of HFpEF patients without pre-existing persistent/permanent AF, which are, however, at an increased risk of concomitant AF,^39^ is completely unknown.

Here, we show an increased Na influx in atrial cardiomyocytes of HFpEF patients (*Figure 1*). Importantly, treatment of the cells with the late I_Na_ inhibitor TTX as well as the specific CaMKII inhibitor AIP reduced Na influx to the level of non-failing cardiomyocytes underscoring the relevance of CaMKII and late I_Na_ for Na dysregulation in our HFpEF myocytes (*Figure 4A* and *B*). This is further corroborated by our patch-clamp data showing an increase in late I_Na_ in atrial cardiomyocytes of HFpEF patients compared to NF patients (*Figure 2D* and *E*), which could be abolished by treatment of the cells with the CaMKII inhibitor AIP (*Figure 5A* and *B*). Moreover, the inhibition of NHE1, another relevant contributor to Na overload in ventricular cardiomyocytes of patients with HFrEF,^40,41^ by its specific inhibitor cariporide could also significantly reduce Na influx (*Figure 4A* and *B*). At first glance, this seems to be in contrast to our recently published data, showing no increase in NHE1 abundance in atrial cardiomyocytes of HFpEF patients (in sinus rhythm).^16^ However, NHE1 activity is also regulated by post-translational modifications, including stimulation by CaMKII-dependent phosphorylation,^42^ which could explain the observed NHE1-mediated Na influx into atrial HFpEF myocytes.

### 4.2 HFpEF is associated with increased CaMKII activity in human atrial cardiomyocytes

Although CaMKII has been reported to improve myofilament passive stiffness by phosphorylating titin,^43^ which may reduce diastolic dysfunction in HFpEF, it is as yet unclear whether CaMKII may also contribute to the HFpEF phenotype through deleterious changes in EC coupling, particularly through the dysregulation of Na homeostasis described above.

While there are no data regarding CaMKII activity in the atria of HFpEF patients or from animal models of HFpEF, Franssen *et al.* previously reported an increase in CaMKII activity in the ventricular myocardium of ZSF1-HFpEF rats.^21^ This is consistent with our results showing an increase in CaMKII activity as well as CaMKII-dependent Na_V_1.5 hyperphosphorylation in human atrial cardiomyocytes of HFpEF patients based on increased CaMKII expression and autophosphorylation at Thr287 (*Figure 2A* and *3*). These findings underscore the relevance of CaMKII for the regulation of Na_V_1.5 in HFpEF. Noteworthy, in a porcine model of AF-induced heart failure, inhibition of CaMKII improved atrial contractile function and attenuated atrial fibrosis, hypertrophy, and apoptosis, pointing to the role of CaMKII for arrhythmia-related atrial remodelling.^44^

### 4.3 Na handling in atrial cardiomyocytes of HFpEF patients is independent of paroxysmal AF, diabetes, or kidney function

In addition to the HFpEF phenotype, several other factors could theoretically explain our experimental results. For example, atrial fibrillation and diabetes are known to be associated with a (CaMKII-dependent) increase in late I_Na_^22,23,44–46^ as well as NHE1 activity.^47,48^ Almost 30% of our HFpEF patient cohort were diagnosed with diabetes and 15% with paroxysmal AF. HbA1c levels were adequately controlled (HbA1c ∼6.0%) making glycaemia-induced alterations in CaMKII-dependent or NHE1-dependent pathways less likely.^46^

Importantly, our multivariate linear regression analyses showed that all these factors do not confound our experimental results and confirm HFpEF as an independent predictor of increased CaMKII expression/auto-phosphorylation, Na influx as well as Na_V_1.5 phosphorylation (by trend). However, only patients with paroxysmal AF and probably less pronounced (and reversible) atrial remodelling due to AF^49,50^ were included in our study. Therefore, we cannot make any statement about patients with persistent or permanent atrial fibrillation.

Arterial hypertension and chronic kidney disease are other known conditions that promote CaMKII-mediated as well as NHE1-mediated cardiac alterations.^51–53^ Consequently, we also included these parameters in our model II of multivariate linear regression without finding a significant correlation.

Surprisingly, NT-proBNP levels (measured at admission) failed to correlate with our experimental readouts. On the one hand, this could be caused by the fact that NT-proBNP levels are highly dynamic and might thus not reflect the severity of chronic atrial remodelling.^54,55^ On the other hand, NT-proBNP levels are not only determined by myocardial wall stress but also influenced by, e.g. age, sex, kidney function, diabetes, and BMI,^56–58^ which probably prevents a correlation between NT-proBNP levels and measurements of disturbed Na homeostasis in our study.

### 4.4 Empagliflozin inhibits increased late I_Na_ and overall Na influx in atrial HFpEF myocytes

Previously, SGLT2i have been shown to directly or indirectly interfere with cardiomyocyte Na handling in patients with HFrEF and animal models of systolic contractile dysfunction.^12–17^ Philippaert and colleagues recently showed an SGLT2i-mediated inhibition of late I_Na_ in failing murine ventricular cardiomyocytes as well as in HEK293T cells expressing mutated human Na_V_1.5 channels and proposed a direct binding of SGLT2i to Na channels by application of molecular docking simulations.^13^ Although the relevance of CaMKII for the regulation of late I_Na_ was not investigated, it is conceivable that CaMKII inhibition contributes to the empagliflozin-mediated effects in their study.

This notion is supported by results from experiments in a mouse model of pressure overload-induced heart failure and in ventricular cardiomyocytes of patients with end-stage HFrEF, where empagliflozin significantly reduced CaMKII activity, which was associated with improved cellular Ca handling as well as acute reduction in [Na]_i_.^12^ Furthermore, in human ventricular cardiomyocytes of patients with aortic stenosis and a consequent HFpEF phenotype, empagliflozin significantly inhibited increased late I_Na_ comparable to AIP, while it was not able to inhibit ATXII- or veratridine-induced late I_Na_.^30^ These findings point to an, at least in part, indirect inhibition of this current via interference with CaMKII signalling.^30^ Similarly, in mouse models of HFpEF, empagliflozin pre-incubation (4 h) but not acute treatment (3 min) was not only capable of reducing late I_Na_ but also cellular proarrhythmia in ventricular cardiomyocytes in a CaMKII-dependent manner.^59,60^

Moreover, in a rat model of HFpEF (ZSF-1 obese) attenuation of left atrial remodelling and cellular arrhythmogenesis by treatment with the SGLT-1/2 inhibitor sotagliflozin was associated with an increased forward mode NCX (Na/Ca-exchanger) activity.^61^ Although neither [Na]_i_ nor Na influx was assessed in that study, the authors suggested that a reduction of initially elevated [Na]_i_ upon sotagliflozin treatment might account for the observed increase in forward mode NCX activity.^61^

In the present study, we show that exposure to empagliflozin significantly reduced the overall Na influx in atrial myocytes of patients with HFpEF, similar to AIP, TTX, and cariporide. Importantly, we also demonstrate inhibition of increased late I_Na_ by treatment with empagliflozin in these cells comparable to the effect of CaMKII inhibition by AIP. Therefore, it is tempting to speculate that empagliflozin may also inhibit CaMKII and resulting CaMKII-dependent Na_V_1.5 regulation in HFpEF. However, our experimental design does not allow us to differentiate between the effects of empagliflozin on CaMKII, late I_Na_, and NHE1. Consequently, we cannot distinguish, whether empagliflozin-mediated direct inhibition of CaMKII results in a decrease in late I_Na_ and/or altered NHE1 function or whether direct inhibition of these sodium handling proteins results in decreased Ca-dependent CaMKII activation by improved cellular Na and Ca handling.

In this regard, it is important to mention that several lines of evidence also suggest a decrease in [Na]_i_ as a consequence of SGLT2i-mediated inhibition of NHE1 in healthy ventricular cardiomyocytes from mice, rats, and rabbits.^14,15,17^ Using docking simulations the Na ^+^ -binding site of NHE1 was identified as a potential binding site of SGLT2i.^15^ Although we could recently reproduce an inhibitory effect of empagliflozin on NHE1 function in human atrial cardiomyocytes (including diseased myocytes),^16^ NHE1 inhibition by SGLT2i and its effect on cellular Na handling is still a matter of debate, since Chung *et al.* were not able to reproduce these results in healthy ventricular cardiomyocytes from mouse, rat and guinea pig hearts.^18^ It is indeed questionable if NHE1 inhibition can decrease [Na]_i_ in healthy cardiomyocytes, where NHE1 activity is low at normal intracellular pH and thus contributes only little to Na loading.^62^ Here, we show that treatment of cardiomyocytes from HFpEF patients with cariporide significantly reduces Na influx. In this regard, it is also important to mention that treatment of atrial cardiomyocytes with empagliflozin and TTX resulted in an even more pronounced inhibition of overall Na influx than treatment with TTX alone (Figure 3*A*), suggesting an effect of empagliflozin beyond late I_Na_ inhibition. Thus, it is conceivable that direct or indirect (via CaMKII) inhibition of NHE1 might contribute to, but not fully explain, reduced Na influx in HFpEF cardiomyocytes upon empagliflozin treatment.

Intriguingly, independent of the precise mechanism, reduction of increased Na influx into atrial HFpEF cardiomyocytes by SGLT2i might be key to understand the cardioprotective effects of these drugs in HFpEF patients.^3^ Increased Na influx via late I_Na_ is centrally involved in the generation of early (EAD) and delayed afterdepolarizations (DAD), the cellular basis for atrial, as well as ventricular arrhythmias.^20,63–67^ Noteworthy, post-hoc analyses of clinical trial data hint at anti-arrhythmic effects of SGLT2i. An explorative analysis of the DECLARE-TIMI 58 trial revealed a 19% reduction in the incidence of atrial fibrillation/atrial flutter in the dapagliflozin group in a high-risk collective of type II diabetic patients.^10^ Furthermore, a meta-analysis of 22 trials including 52 115 patients suggests that SGLT2i reduces the risk of atrial fibrillation, embolic stroke, and ventricular tachycardia.^11^

### 4.5 Limitations

As mentioned above, our experimental design does not allow us to distinguish between the effects of empagliflozin on late I_Na_, CaMKII, and NHE1. This limitation is inherent to experiments in adult human cardiomyocytes that are not easily accessible for targeted genetic modifications. Furthermore, an indirect approach by estimation of an additive effect of specific inhibitors requires a lengthy and potentially injurious protocol, which most of the isolated human atrial cardiomyocytes would not sustain. Consequently, our approach is a trade-off between the investigation of important pathophysiological alterations in human HFpEF cardiomyocytes and practicability.

In addition, in this study, we used the non-ratiometric Na-dye ASANTE Na Green-2 AM for the measurement of Na influx. Although this dye is very useful for assessing changes in fluorescence (and therefore Na concentration) over time, it is not suitable for accurately measuring Na concentration quantitatively. However, the advantage of this dye is the short incubation time, which is critical when working with freshly isolated adult human cardiomyocytes that tend to deteriorate in cell quality and viability quite rapidly.

Another potential limitation is the fact that the pathophysiological alterations observed in atrial cardiomyocytes cannot unreservedly be transferred to ventricular myocytes. However, especially for potential anti-arrhythmic effects of SGLT2i (e.g. in regards to atrial fibrillation), understanding Na handling in human atrial HFpEF cardiomyocytes might be crucial.

A technical limitation of our study is the application of BDM, which was used to enhance cell viability during isolation. BDM is a reversible inhibitor of myosin ATPase, but has been shown to reversibly inhibit Ca currents in the adult rat cervical ganglion^68^ and induce Ca release from the canine cardiac sarcoplasmic reticulum.^69^ Although we extensively washed out BDM before starting data acquisition, we cannot completely exclude a possible influence of BDM on intracellular Na and Ca handling.

## 5. Conclusion

Here, we demonstrate for the first time an increased Na influx in human cardiomyocytes from HFpEF patients that may be due, at least in part, to CaMKII-mediated Na_V_1.5 hyperphosphorylation with consequently increased late I_Na_ as well as increased NHE1 activity. Importantly, the increased Na influx could be normalized by the SGLT2i empagliflozin, which was previously shown to inhibit CaMKII, late I_Na_ and NHE1.^12,14–16,30,40,59,60^ However, the specific target through which SGLT2i affect Na handling in atrial HFpEF cardiomyocytes remains elusive and requires further investigation, as it may have helped to reduce the combined risk of HHF and cardiovascular death in a recent landmark clinical trial in HFpEF patients.^3^

**Translational perspective**

Treatment strategies for patients with HFpEF are limited, not least because the underlying mechanisms are poorly understood. Here, we propose a role for increased CaMKII-dependent and late I_Na_-mediated Na influx in atrial electrical remodelling in human HFpEF. Alterations in Na handling are known to promote diastolic dysfunction and arrhythmogenesis, both key features of HFpEF. Here, we demonstrate for the first time that treatment with an SGLT2i—the only class of drugs to date shown to reduce the risk of hospitalization for heart failure and cardiovascular death in HFpEF patients—leads to a reduction in Na influx in atrial cardiomyocytes of HFpEF patients, which may contribute to the improved clinical outcome.

## Supplementary Material

cvae095_Supplementary_Data

## Data Availability

The authors declare that all method protocols used in this study are made available for any researcher upon request. To exclude the possibility of unintentionally sharing private patient information, patient data can only be made available after informed consent about a specific request has been given by each patient.
