# Supplementary material for: Empagliflozin inhibits increased Na influx in atrial cardiomyocytes of patients with HFpEF
Source: Cardiovasc Res. 2024 May 10;120(9):999–1010. doi: 10.1093/cvr/cvae095 (PMC11288740; doi:10.1093/cvr/cvae095)
Supplement: cvae095_Supplementary_Data [file cvae095_supplementary_data.zip › Supplementary Figures_Tables_revision_SW_MT_final.docx]

**Supplementary Figures/Tables**

**Empagliflozin inhibits increased Na influx in atrial cardiomyocytes of patients with HFpEF**

M. Trum^1*^, J. Riechel^1*^, E. Schollmeier^1^, S. Lebek^1^, P. Hegner^1^, K. Reuthner^1^, S. Heers^1^, K. Keller^1^, M. Wester^1^, S. Klatt^1^, N. Hamdani^2^, Z. Provaznik^3^, C. Schmid^3^, L.S. Maier^1^, M. Arzt^1^, S. Wagner^1^

^1^Department of Internal Medicine II, University Hospital Regensburg, Regensburg, Germany

^2^Department of Cellular and Translational Physiology, Ruhr-University Bochum, Bochum, Germany

^3^Department of Cardiothoracic Surgery, University Hospital Regensburg, Regensburg, Germany

***** these authors contributed equally

**Short title:** Empagliflozin inhibits Na influx in HFpEF

**Article type:** Original article

**Corresponding author:**

Prof. Dr. med. Stefan Wagner

Department of Internal Medicine II

University Hospital Regensburg

Franz-Josef-Strauß-Allee 11

93053 Regensburg

Germany

Phone: +49-941-9447206

Fax: +49-941-9447339

Email: stefan.wagner@ukr.de

**
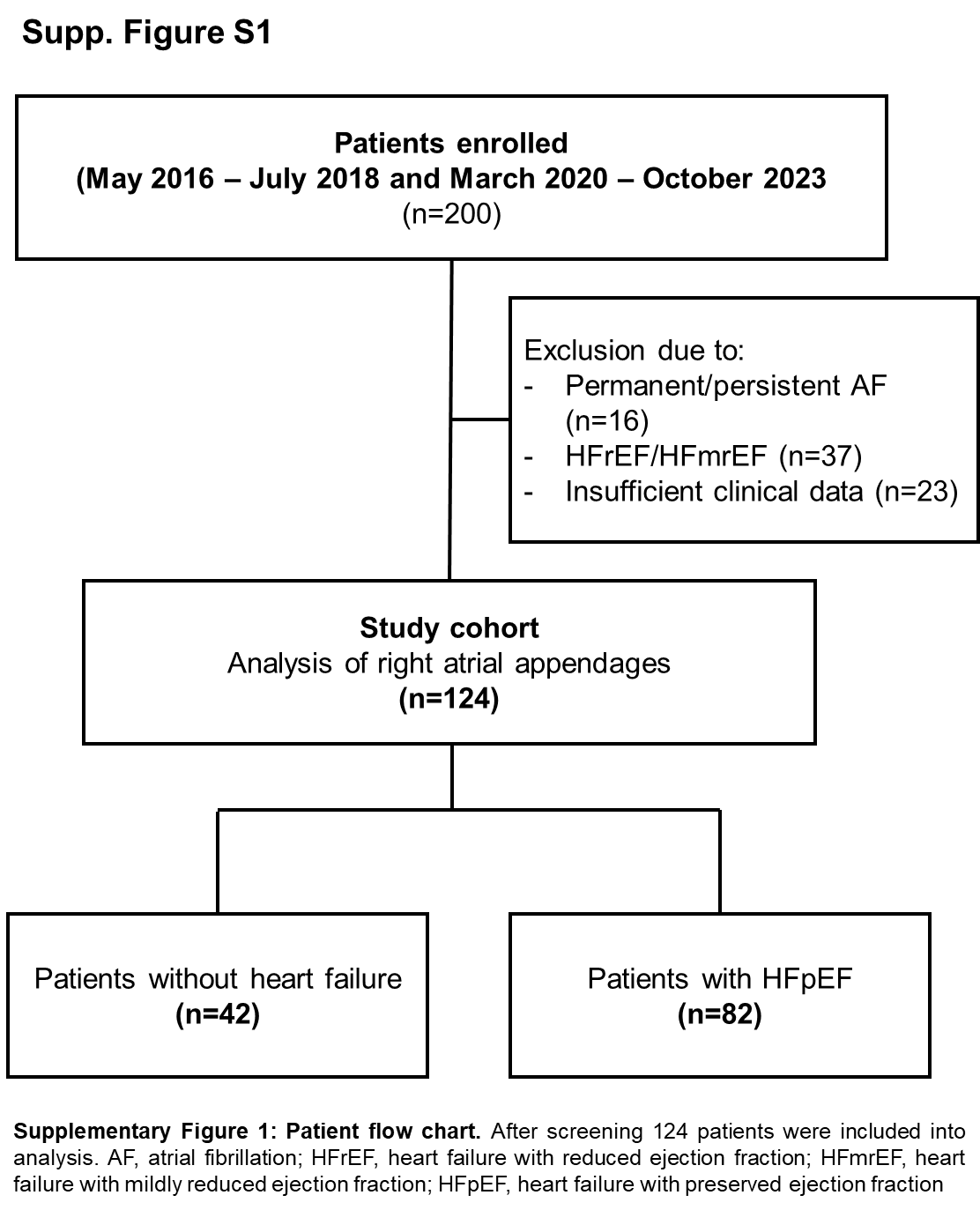
**

**
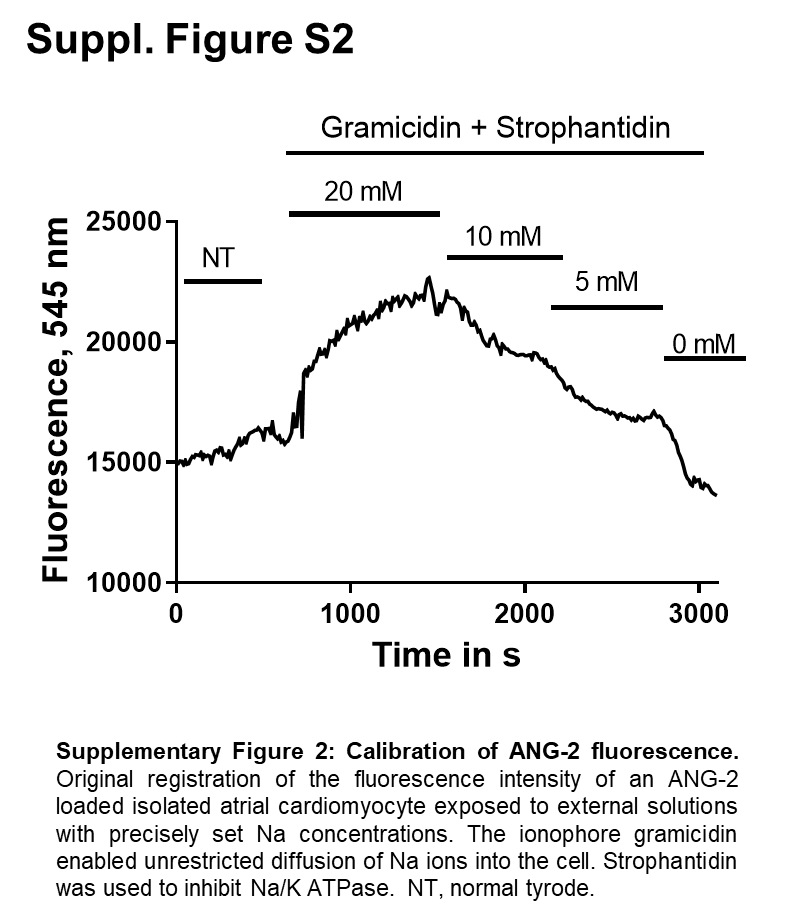
**

**
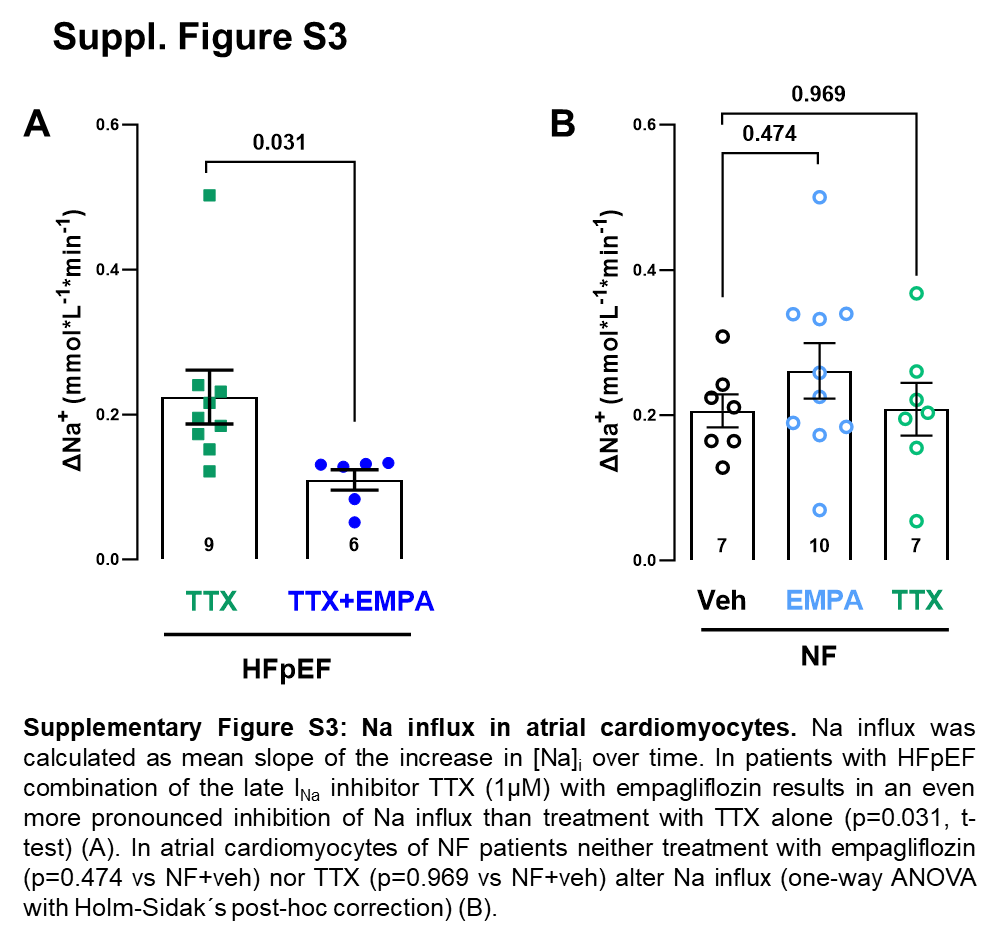
**

| Supplementary table S1. Linear Regression analysis for pNa_V_1.5 | | | | | | |
| --- | --- | --- | --- | --- | --- | --- |
|  | Simple Linear Regression Analysis | | Multiple Linear Regression Analysis | | | |
|  |  | | Model I | R^2^ **0.242** | Model II | R^2^ **0.368** |
| Variable | **B (95% CI)** | **P value** | **B (95% CI)** | **P value** | **B (95% CI)** | **P value** |
| HFpEF | 0.5493 (0.0951; 1.0034) | **0.019** | 0.4948 (0.0049; 0.9847) | **0.048** | 0.5589 (-0.1367; 1.2544) | 0.107 |
| Age | 0.0135 (-0.0158; 0.0427) | 0.353 | 0.0042 (-0.0244; 0.0327) | 0.766 | 0.0155 (-0.0194; 0.0504) | 0.359 |
| Male gender | Omitted, collinearity |  | Omitted, collinearity |  | Omitted, collinearity |  |
| BMI | -0.0275 (-0.0701; 0.0152) | 0.196 | -0.0216 (-0.0622; 0.0190) | 0.283 | -0.0050 (-0.0606; 0.0507) | 0.852 |
| AF | 0.2129 (-0.4069; 0.8326) | 0.486 |  |  | 0.0437 (-0.7769; 0.8643) | 0.911 |
| Diabetes | -0.3880 (-0.8480; 0.0720) | 0.095 |  |  | -0.2598 (-0.9047; 0.3850) | 0.404 |
| Art. Hypertension | -0.0649 (-0.7720; 0.6421) | 0.851 |  |  | -0.3666 (-1.4635; 0.7302) | 0.487 |
| NT-proBNP | 0.00003 (-0.00007; 0.0001) | 0.535 |  |  | -2.97x10^-6^ (-0.0002; 0.0002) | 0.968 |
| eGFR | -0.0037 (-0.0136; 0.0062) | 0.446 |  |  | 0.0038 (-0.0126; 0.0201) | 0.630 |

Model I accounts for age, gender and BMI. Model II accounts for age, male gender, BMI, AF, diabetes, arterial hypertension, NT-proBNP and eGFR. Of note, male gender was omitted from this analysis since all patients in this analysis were coincidentally male. Abbreviations: HFpEF: heart failure with preserved ejection fraction; BMI: body mass index (in kg/m^2^); AF: atrial fibrillation; NT-proBNP: N-terminal pro-brain natriuretic peptide (in pg/ml); eGFR: estimated glomerular filtration rate (in ml/min/1.73m^2^).

| Supplementary table S2. Linear Regression analysis for CaMKII Expression | | | | | | |
| --- | --- | --- | --- | --- | --- | --- |
|  | Simple Linear Regression Analysis | | Multiple Linear Regression Analysis | | | |
|  |  | | Model I | R^2^ **0.156** | Model II | R^2^ **0.255** |
| Variable | **B (95% CI)** | **P value** | **B (95% CI)** | **P value** | **B (95% CI)** | **P value** |
| HFpEF | 0.3540 (0.1030; 0.6050) | **0.007** | 0.3820 (0.0788; 0.6852) | **0.015** | 0.4994 (0.1497; 0.8491) | **0.006** |
| Age | 0.0051 (-0.0100; 0.0199) | 0.495 | -0.0028 (-0.0191; 0.0135) | 0.730 | -0.0104 (-0.0311; 0.0102) | 0.312 |
| Male gender | 0.1066 (-0.2088; 0.4219) | 0.500 | -0.0467 (-0.3823; 0.2889) | 0.780 | -0.0415 (-0.4210; 0.3380) | 0.826 |
| BMI | 0.0102 (-0.0214; 0.0419) | 0.518 | 0.0087 (-0.0223; 0.0396) | 0.575 | -0.0023 (-0.0399; 0.0353) | 0.901 |
| AF | -0.0845 (-0.4891; 0.3202) | 0.676 |  |  | -0.0842 (-0.6272; 0.4588) | 0.755 |
| Diabetes | 0.1331 (-0.1358; 0.4019) | 0.324 |  |  | 0.1003 (-0.2393; 0.4399) | 0.553 |
| Art. Hypertension | -0.0642 (-0.5757; 0.4474) | 0.802 |  |  | -0.4801 (-1.1425; 0.1823) | 0.150 |
| NT-proBNP | -0.00002 (-0.0001; 0.00005) | 0.515 |  |  | -0.00009 (-0.0002; 0.00003) | 0.145 |
| eGFR | -0.0025 (-0.0081; 0.0031) | 0.378 |  |  | -0.0027 (-0.0113; 0.0059) | 0.526 |

Model I accounts for age, gender and BMI. Model II accounts for age, male gender, BMI, AF, diabetes, arterial hypertension, NT-proBNP and eGFR. Abbreviations: HFpEF: heart failure with preserved ejection fraction; BMI: body mass index (in kg/m^2^); AF: atrial fibrillation; NT-proBNP: N-terminal pro-brain natriuretic peptide (in pg/ml); eGFR: estimated glomerular filtration rate (in ml/min/1.73m^2^).

| Supplementary table S3. Linear Regression analysis for pCaMKII | | | | | | |
| --- | --- | --- | --- | --- | --- | --- |
|  | Simple Linear Regression Analysis | | Multiple Linear Regression Analysis | | | |
|  |  | | Model I | R^2^ **0.462** | Model II | R^2^ **0.702** |
| Variable | **B (95% CI)** | **P value** | **B (95% CI)** | **P value** | **B (95% CI)** | **P value** |
| HFpEF | 0.3529 (0.1701; 0.5357) | **0.001** | 0.2945 (0.0744; 0.5147) | **0.011** | 0.3392 (0.1223; 0.5562) | **0.005** |
| Age | 0.0123 (-0.0003; 0.0248) | 0.055 | 0.0067 (-0.0058; 0.0192) | 0.273 | -0.0067 (-0.0240; 0.0107) | 0.421 |
| Male gender | 0.1452(-0.1263; 0.4167) | 0.279 | 0.0652 (-0.1772; 0.3077) | 0.580 | -0.0520 (-0.3319; 0.2278) | 0.694 |
| BMI | -0.0042 (-0.0333; 0.0248) | 0.765 | -0.0002 (-0.0245; 0.0242) | 0.990 | -0.0121 (-0.0408; 0.0167) | 0.380 |
| AF | 0.3140 (-0.0717; 0.6997) | 0.105 |  |  | 0.1763 (-0.2139; 0.5664) | 0.347 |
| Diabetes | 0.1779 (-0.0586; 0.4145) | 0.133 |  |  | 0.2638 (-0.0088 ;0.5364) | 0.057 |
| Art. Hypertension | -0.0216 (-0.5885; 0.5453) | 0.938 |  |  | -0.0573 (-0.6028; 0.4881) | 0.824 |
| NT-proBNP | 0.0002 (-7.9x10^-6^; 0.0005) | 0.058 |  |  | 0.0001 (-0.0002; 0.0004) | 0.417 |
| eGFR | -0.0060 (-0.0111; -0.0010) | **0.021** |  |  | -0.0024 (-0.0088; 0.0039) | 0.422 |

Model I accounts for age, gender and BMI. Model II accounts for age, male gender, BMI, AF, diabetes, arterial hypertension, NT-proBNP and eGFR. Abbreviations: HFpEF: heart failure with preserved ejection fraction; BMI: body mass index (in kg/m^2^); AF: atrial fibrillation; NT-proBNP: N-terminal pro-brain natriuretic peptide (in pg/ml); eGFR: estimated glomerular filtration rate (in ml/min/1.73m^2^).
