# Supplementary material for: Empagliflozin inhibits increased Na influx in atrial cardiomyocytes of patients with HFpEF
Source: Cardiovasc Res. 2024 May 10;120(9):999–1010. doi: 10.1093/cvr/cvae095 (PMC11288740; doi:10.1093/cvr/cvae095)

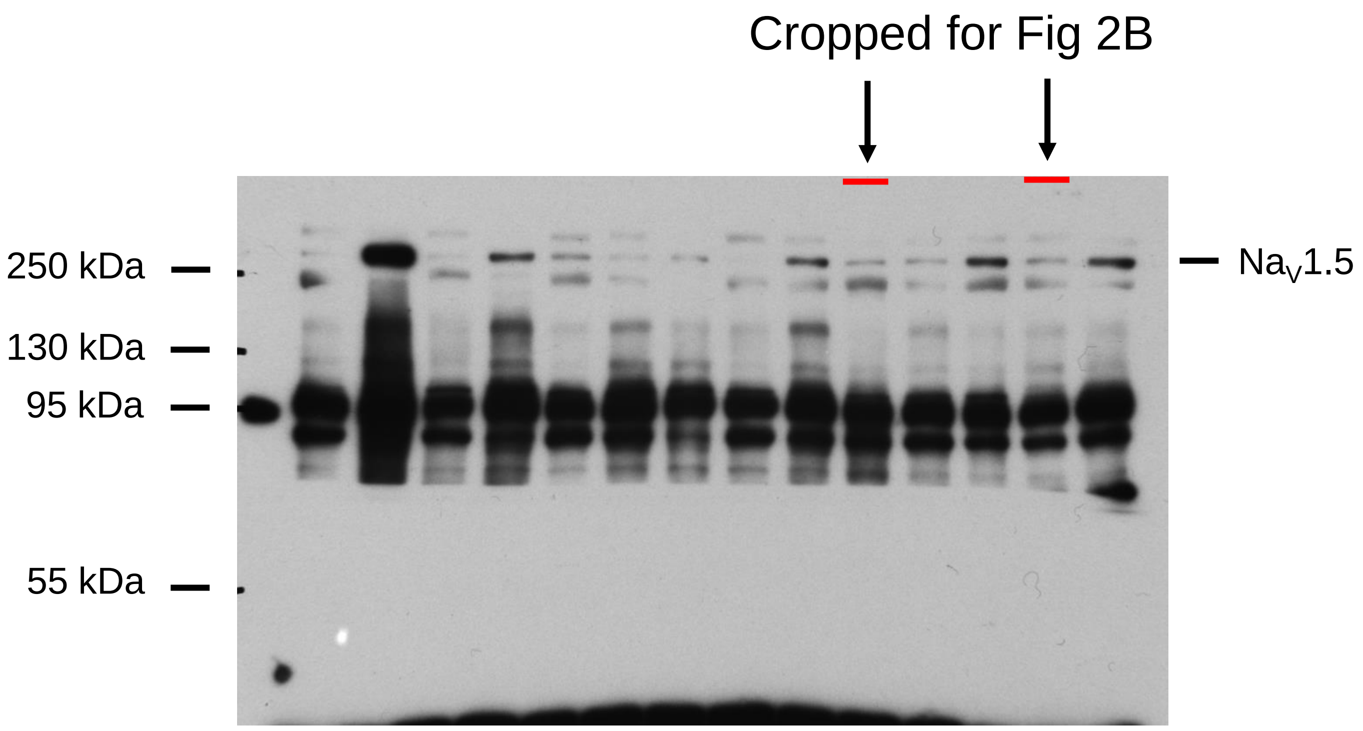
Cropped for Fig 2B
250 kDa
130 kDa
95 kDa
55 kDa
NaV1.5

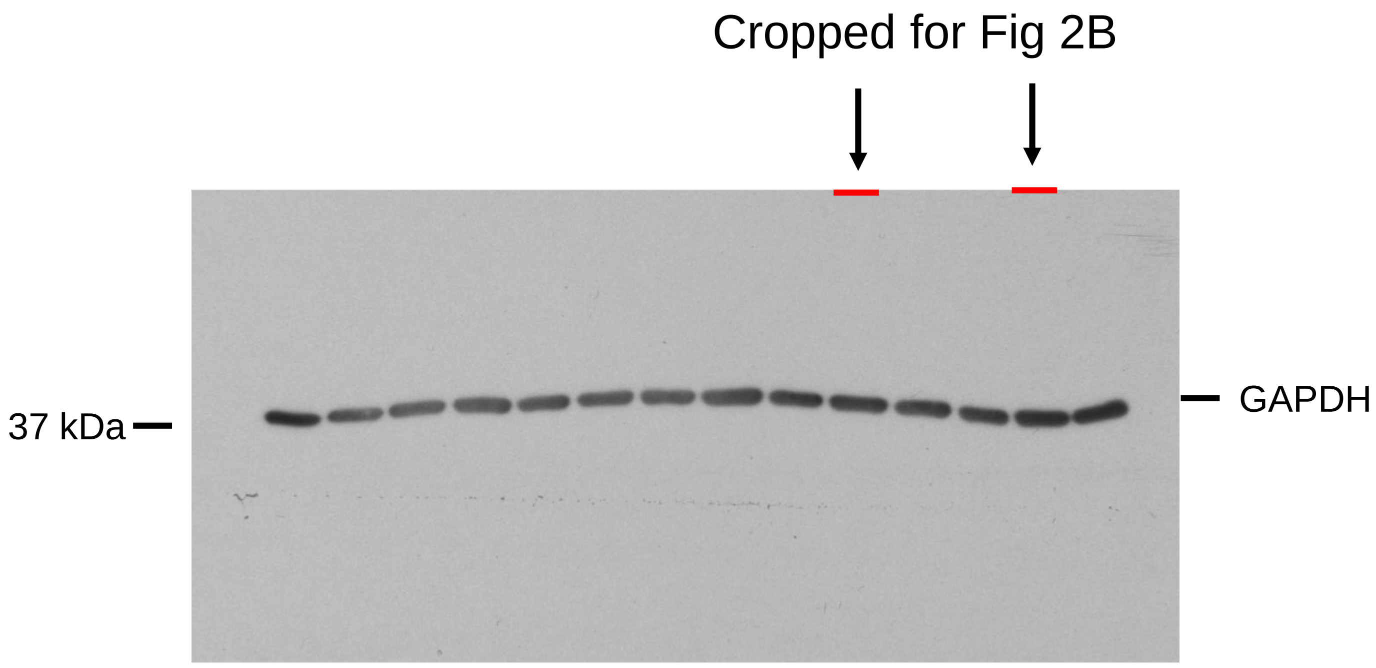
Cropped for Fig 2B
37 kDa
GAPDH

Cropped for Fig 2C

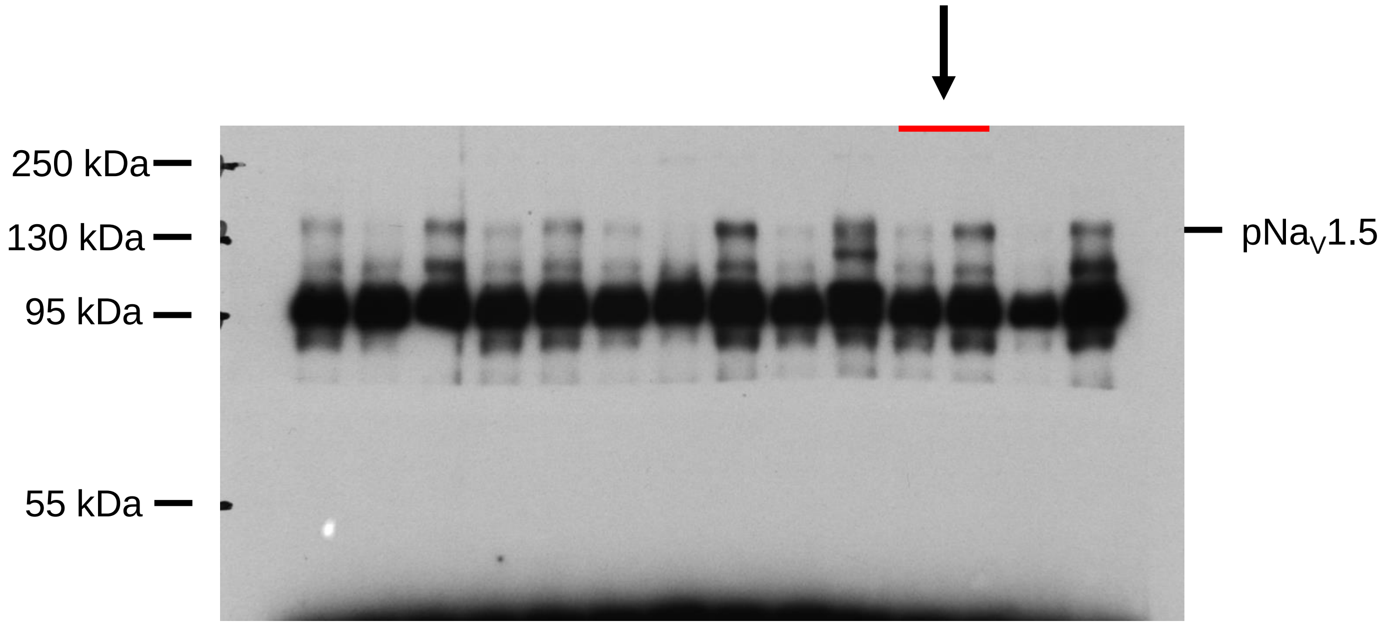

Cropped for Fig 2C

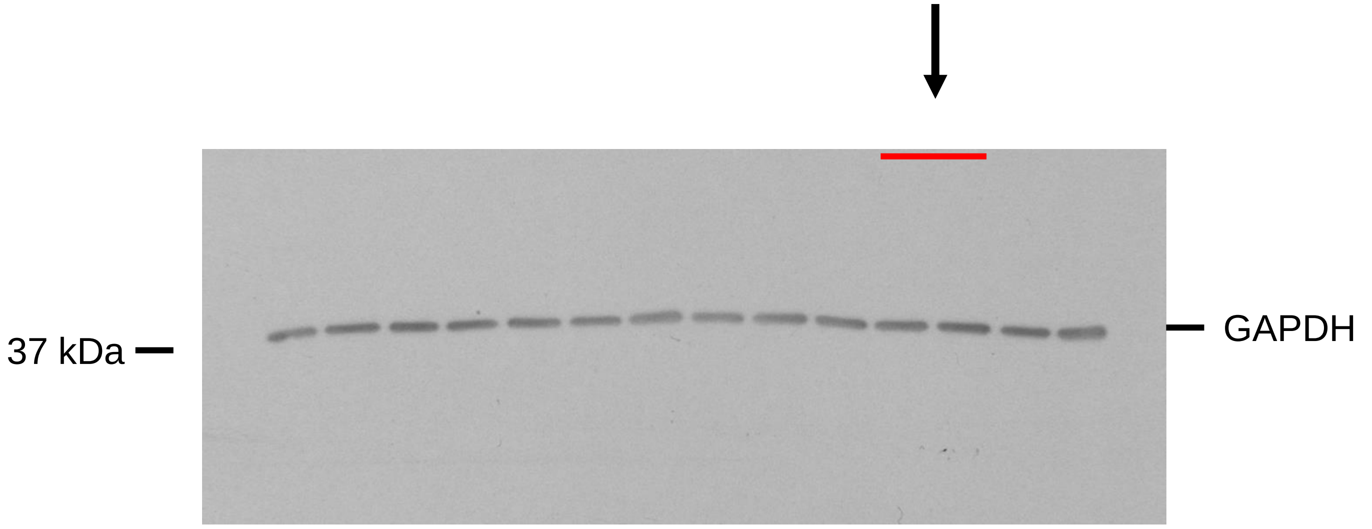

Cropped for Fig 3A

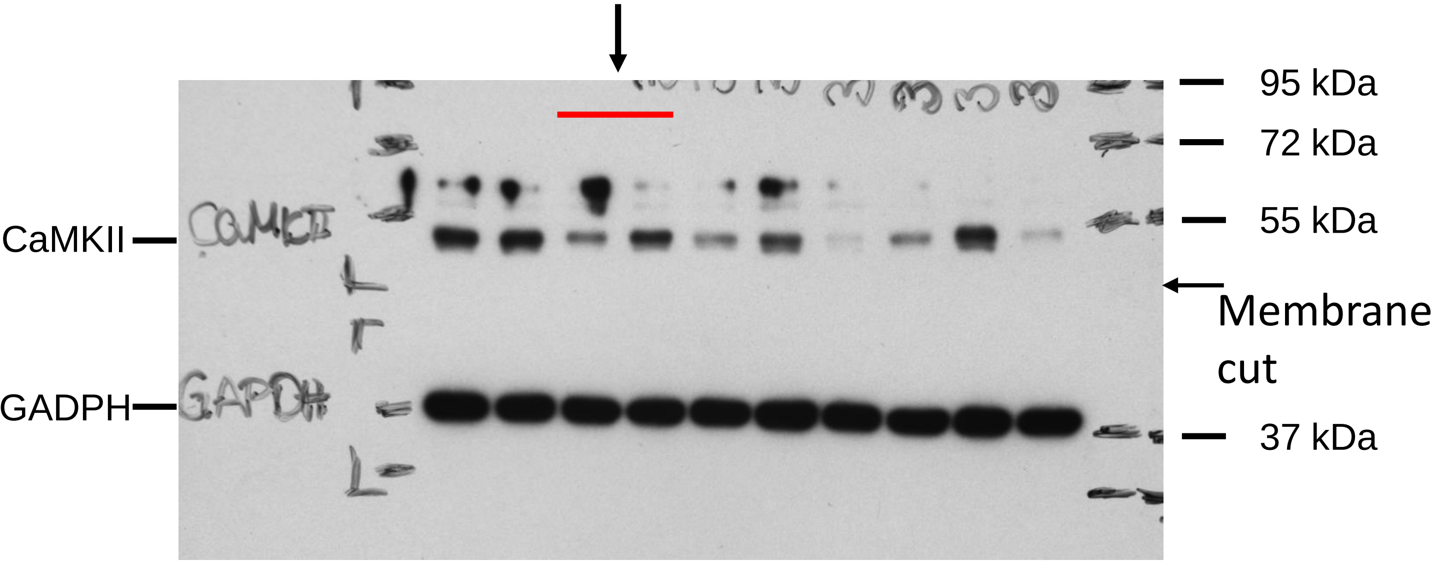

Cropped for Fig 3A

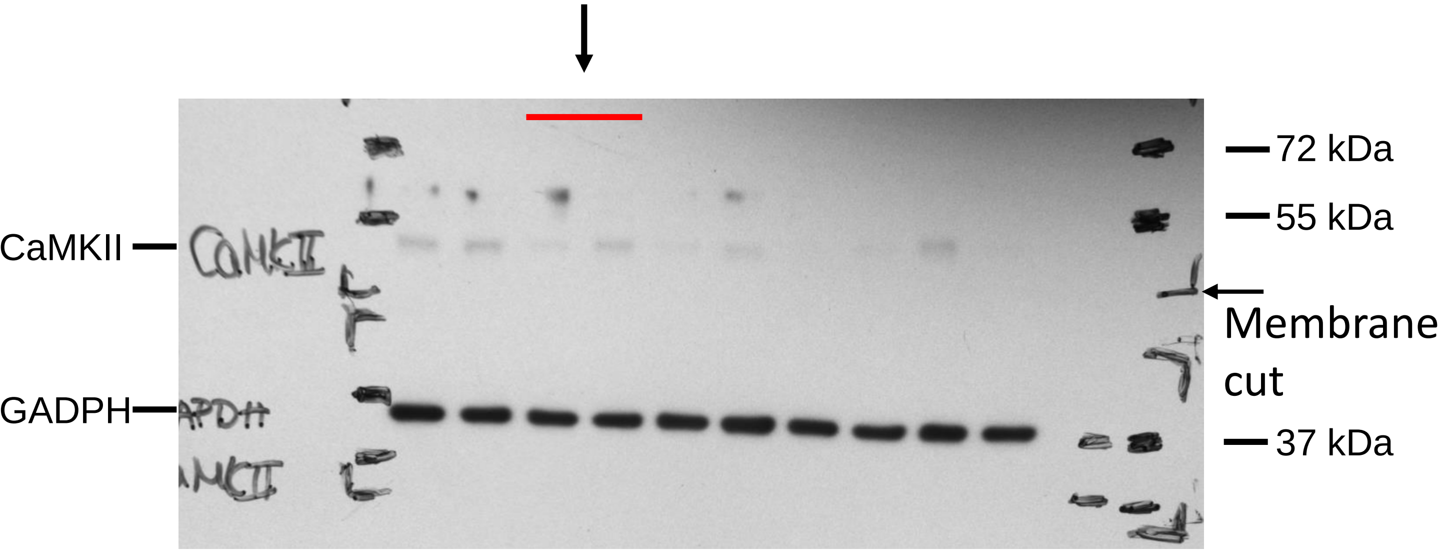

Cropped for Fig 3B

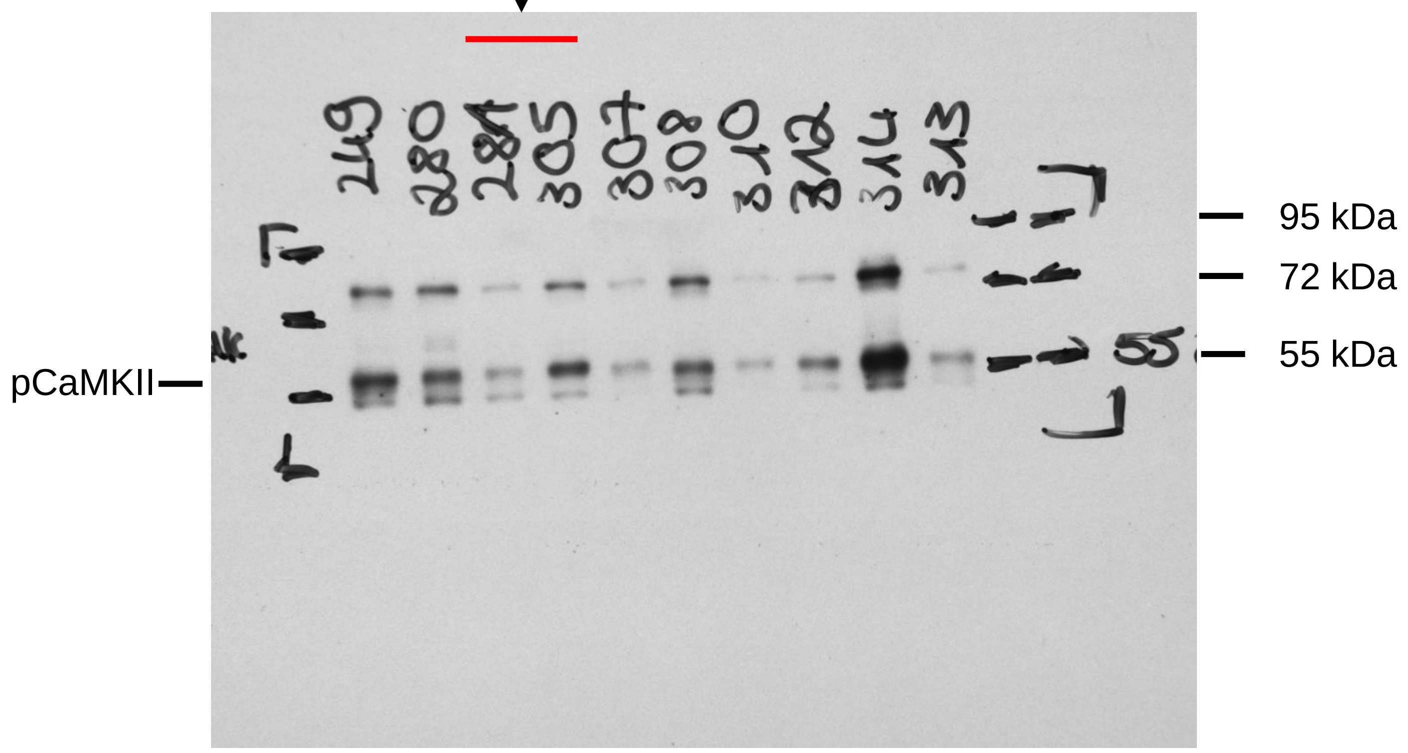

Cropped for Fig 3B

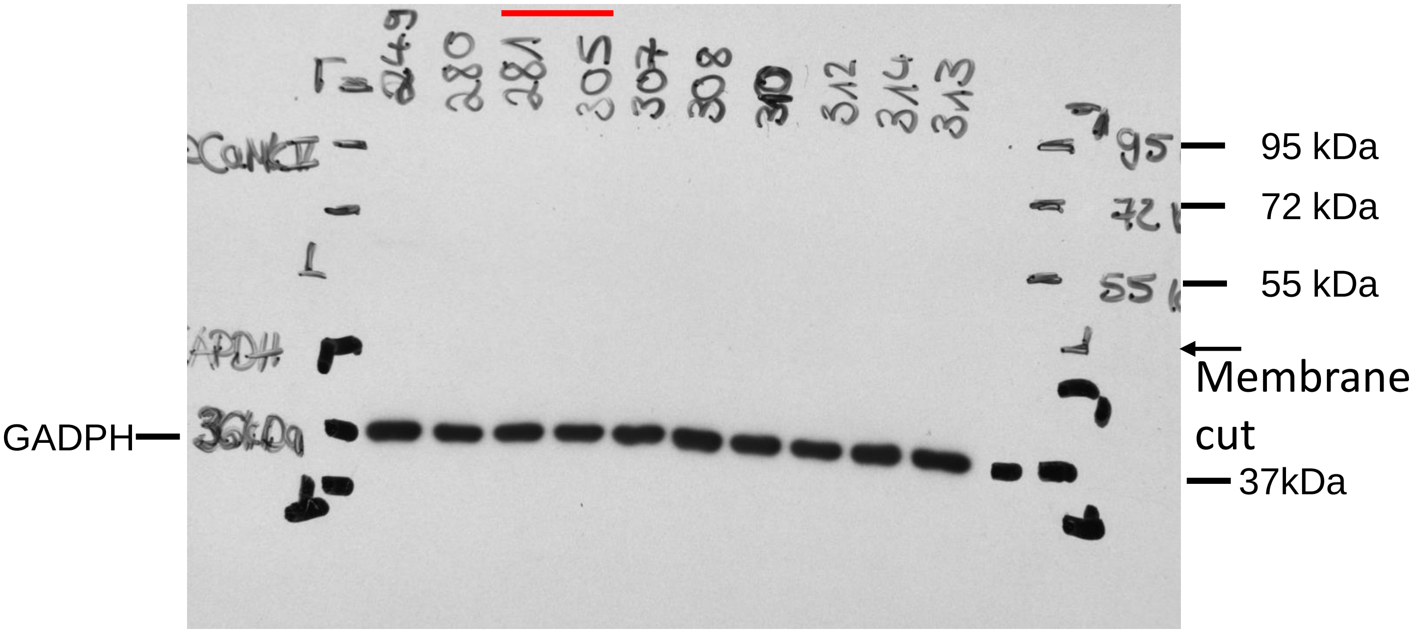

Supplement: cvae095_Supplementary_Data [file cvae095_supplementary_data.zip › Uncropped_gels.pdf]
